# Variation and evolution of polyadenylation profiles in sauropsid mitochondrial mRNAs as deduced from the high-throughput RNA sequencing

**DOI:** 10.1186/s12864-017-4080-0

**Published:** 2017-08-29

**Authors:** Yao Sun, Masaki Kurisaki, Yasuyuki Hashiguchi, Yoshinori Kumazawa

**Affiliations:** 1 Department of Information and Basic Science and Research Center for Biological Diversity, Graduate School of Natural Sciences, Nagoya City University, 1 Yamanohata, Mizuho-cho, Mizuho-ku, Nagoya 467-8501 Japan; 2 Department of Biology, Osaka Medical College, Takatsuki, Japan

**Keywords:** Transcription, RNA processing, PolyA, Gene overlap, Gene rearrangement

## Abstract

**Background:**

Genes encoded in vertebrate mitochondrial DNAs are transcribed as a polycistronic transcript for both strands, which is later processed into individual mRNAs, rRNAs and tRNAs, followed by modifications, such as polyadenylation at the 3′ end of mRNAs. Although mechanisms of the mitochondrial transcription and RNA processing have been extensively studied using some model organisms, structural variability of mitochondrial mRNAs across different groups of vertebrates is poorly understood. We conducted the high-throughput RNA sequencing to identify major polyadenylation sites for mitochondrial mRNAs in the Japanese grass lizard, *Takydromus tachydromoides* and compared the polyadenylation profiles with those identified similarly for 23 tetrapod species, featuring sauropsid taxa (reptiles and birds).

**Results:**

As compared to the human, a major polyadenylation site for the NADH dehydrogenase subunit 5 mRNA of the grass lizard was located much closer to its stop codon, resulting in considerable truncation of the 3′ untranslated region for the mRNA. Among the other sauropsid taxa, several distinct polyadenylation profiles from the human counterpart were found for different mRNAs. They included various truncations of the 3′ untranslated region for NADH dehydrogenase subunit 5 mRNA in four taxa, bird-specific polyadenylation of the light-strand-transcribed NADH dehydrogenase subunit 6 mRNA, and the combination of the ATP synthase subunit 8/6 mRNA with a neighboring mRNA into a tricistronic mRNA in the side-necked turtle *Pelusios castaneus*. In the last case of *P. castaneus*, as well as another example for NADH dehydrogenase subunit 1 mRNAs of some birds, the association between the polyadenylation site change and the gene overlap was highlighted. The variations in the polyadenylation profile were suggested to have arisen repeatedly in diverse sauropsid lineages. Some of them likely occurred in response to gene rearrangements in the mitochondrial DNA but the others not.

**Conclusions:**

These results demonstrate structural variability of mitochondrial mRNAs in sauropsids. The efficient and comprehensive characterization of the mitochondrial mRNAs will contribute to broaden our understanding of their structural and functional evolution.

**Electronic supplementary material:**

The online version of this article (10.1186/s12864-017-4080-0) contains supplementary material, which is available to authorized users.

## Background

**Fig. 1:**
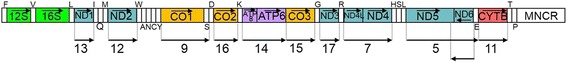
The human mtDNA and its mRNAs. The double-stranded circular mtDNA of the human [1] is linearly expressed and genes contained therein are shown as columns that do not accurately reflect their actual sizes. Gene abbreviations are the following: 12S and 16S, 12S and 16S rRNAs (green columns); ND1–6 and 4 L, NADH dehydrogenase subunits 1–6 and 4 L (blue columns); CO1–3, cytochrome oxidase subunits I-III (orange columns); ATP6 and ATP8, ATP synthase subunits 6 and 8 (violet columns); and CYTB, cytochrome *b* (red column). Transfer RNA genes are indicated as corresponding one-letter amino acids and the major non-coding region is abbreviated as MNCR. The arrows show the encoded direction of protein and rRNA genes. Transfer RNA genes over the column stand for H-strand-encoded ones, whereas those under the column show L-strand-encoded ones. Ranges for H-strand-transcribed mRNAs [7, 34] are shown by horizontal arrows with numbers in the original literature. A range for non-polyadenylated ND6 mRNA [11] is also shown by a reverse horizontal arrow

Mitochondria DNAs (mtDNAs) in vertebrates are double-stranded circular DNAs that are approximately 17kbp in size. They contain intronless genes for 2 rRNAs, 22 tRNAs, 13 respiratory proteins (i.e., cytochrome oxidase subunits I-III, CO1–3; NADH dehydrogenase subunits 1–6 and 4 L, ND1–6 and 4 L; ATP synthase subunits 6 and 8, ATP6 and 8; and cytochrome *b*, CYTB) together with a major noncoding region (MNCR) or a control region which has a regulatory function for replication and transcription [1, 2]. Gene arrangements of vertebrate mtDNAs are relatively conserved and a typical mtDNA gene arrangement found for the human mtDNA [1] (see Fig. 1) is conserved in many species of fish, amphibians, reptiles and mammals. However, gene rearrangements in mtDNAs, as well as the duplication of the MNCR, have been reported from diverse vertebrate species ([2–4] and refs. Therein).

In this typical arrangement of genes, all protein genes except for ND6, 2 rRNA genes, and 14 out of the 22 tRNA genes are encoded by the heavy strand (H-strand), while the ND6 and eight remaining tRNA genes are encoded by the light strand (L-strand). In the late twentieth century, gene expression of mtDNAs was intensively investigated using some model organisms (e.g., human and mouse). It was found that transcription of mitochondrial genes is initiated from within the MNCR in both directions [5, 6]. Polycistronic precursor RNAs for both strands are processed at the 5′ and 3′ ends of 22 interspersed tRNAs to release individual tRNAs, rRNAs and mRNAs (tRNA punctuation model; [7])(see Fig. 1). Subsequently, polyadenylation occurs at the 3′ terminus of mRNAs, and a CCA sequence is added to the 3′ end of tRNAs. Protein genes encoded in a mtDNA often do not have a complete stop codon at their 3′ ends, and addition of the polyA tail to the 3′ end of processed mRNAs generates a stop codon, such as UAA [1].

Polyadenylation of mRNA is an important process in gene expression. In eukaryotes, a polyA tail works for stabilization of mRNA, transportation from nucleus to cytosol, and promotion of translational initiation whereas the polyadenylation stimulates RNA degradation in bacteria and chloroplasts [8, 9]. PolyA sequences attached to the 3′ end of mammalian mitochondrial mRNAs (mt-mRNAs) lacking the cap structure and long 5′ untranslated region (UTR) sequences are approximately 45-nt long, considerably shorter than those for nuclear-encoded mRNAs [10]. As exceptions, the human ND5 mRNA has even shorter (<10 nt) polyA tails and the human ND6 mRNA was reported to lack the polyadenylation at their heterogeneous 3′ termini [10–12].

Although some enzymes, such as the mitochondrial polyA polymerase and polynucleotide phosphorylase, have been shown to regulate the polyadenylation, regulatory mechanisms in the control of polyadenylation/deadenylation and stabilization/degradation of polyadenylated RNAs are not fully understood albeit with many modern investigations ([9, 13–15] and refs. Therein). In addition, evolutionary conservation in mechanisms of the transcription and RNA processing in non-mammalian vertebrate mitochondria is not very clear. Sauropsids (reptiles and birds) include diverse lineages that possess a variety of mtDNA gene rearrangements ([3, 4] and refs. Therein). These gene rearrangements may have created structural variability of mt-mRNAs in sauropsid taxa.

In this study, we attempted to characterize polyadenylation profiles in mt-mRNAs using the high-throughput RNA sequencing (RNA-Seq) technology to gain new insights into the structural diversity and evolution of mt-mRNAs. The high-throughput sequencing is a technique which can parallelize the sequencing process, producing tens of millions of sequences concurrently [16]. To the best of our knowledge, the high-throughput RNA-Seq has been applied to the characterization of mt-mRNAs in a limited number of vertebrate taxa: the human (see, e.g., [11]), bank vole [17], and codfishes [18]. Here, we conducted the high-throughput RNA-Seq to identify major polyadenylation sites for mt-mRNAs in the Japanese grass lizard, *Takydromus tachydromoides*. We compared the results with those for other species and discussed the timing and mechanisms for changes in the polyadenylation profiles during the evolution of sauropsids.

## Methods

### RNA sequencing

The Japanese grass lizard (*Takydromus tachydromoides*) used for experiments was collected at Chikusa-ku, Nagoya, Japan in 2012. In sacrificing the animal, we employed the decapitation as a physical euthanasic procedure based on the permission by the Animal Experiment Committee of Nagoya City University (Permit No. H21N-02). A mirVana miRNA Isolation Kit (Life Technologies) was used to extract total RNA from fresh liver of *T. tachydromoides*. The extracted RNA was quantitated with NanoVue Plus (GE Healthcare Life Sciences), and its intactness was confirmed with the Agilent 2100 BioAnalyer (Agilent Technologies). A TruSeq RNA Sample Preparation Kit (Illumina) was used to prepare the high-throughput sequencing library. Briefly, enrichment of polyA-containing RNA from the total RNA was conducted with the oligo-dT beads. After the polyA-containing RNA was cut randomly with cation and heat, 1st and 2nd strand cDNA synthesis was carried out with random hexamers. After the end repair, index adaptor sequences were ligated to both ends. The ligate was amplified by the bridge PCR and amplified products were purified with AMPure XP beads (Beckman Coulter) that removed short constructs with less than 100-bp inserts. The prepared samples were run for the paired-end 101-bp sequencing with the Illumina HiSeq™ 2000 Sequencer.

We also downloaded Illumina-based RNA-Seq data for other species from the NCBI SRA database with the accession numbers shown in Table 1. Many of these RNA-Seq data were reported by McGaugh et al. [19] and Wu et al. [20]. Downloaded sra files were converted to two fastq files corresponding to forward and reverse paired-end reads, using the fastq-dump in the NCBI SRA toolkit. Remaining adaptor sequences were removed from the reads using Cutadapt [21] by the default conditions.

**Table 1:** RNA-Seq data analyzed in this study

Higher taxa	Family	Species	organ	SRA Accession No.	read length (bp)^a^	RNA-Seq total reads^b^	mtDNA-derived reads	polyA reads^c^	Accession No. of reference mtDNA^d^
Squamata Lacertilia	Lacertidae	*Takydromus tachydromoides*	liver	DRR072216	101	53,655,734	3,627,027	8795	LC101816
	Scincidae	*Scincella lateralis*	liver	SRR629642	100	91,047,166	5,473,971	3035	KU646826
	Eublepharidae	*Eublepharis macularius*	liver	SRR629643	100	70,002,306	4,146,912	2889	AB738955
	Shinisauridae	*Shinisaurus crocodilurus*	liver	DRR034612	151	19,549,612	1,374,172	1280	AB080274
	Agamidae	*Pogona vitticeps*	liver	SRR629641	100	87,625,132	8,515,182	6198	AB166795
	Agamidae	*Phrynocephalus przewalskii*	mixed tissue	SRR1298770	100	111,576,922	8,671,591	2155	NC_022719
	Iguanidae	*Sceloporus undulatus*	liver	SRR629640	100	71,604,846	4,067,660	3333	AB079242
Squamata Serpentes	Viperidae	*Agkistrodon piscivorus*	liver	SRR629645	100	75,023,884	8,161,006	3236	DQ523161
	Colubridae	*Pantherophis guttatus*	mixed tissue	ERR216308	100	27,504,812	999,617	700	AM236349
	Xenopeltidae	*Xenopeltis unicolor*	liver	SRR629647	100	89,792,506	5,340,226	2728	AB179620
Testudines	Chelydridae	*Chelydra serpentina*	liver	SRR629521	100	67,455,518	2,811,802	1026	EF122793
	Emydidae	*Terrapene carolina*	liver	SRR629650	100	88,575,286	6,905,832	4410	-
	Kinosternidae	*Sternotherus odoratus*	liver	SRR629648	100	95,375,174	2,977,994	1985	NC_017607
	Pelomeducidae	*Pelusios castaneus*	liver	SRR629649	100	90,326,648	8,875,110	4853	KC692463
Crocodylia	Alligatoridae	*Alligator mississippiensis*	liver	SRR629636	100	72,260,274	3,303,351	4719	Y13113
Aves	Phasianidae	*Gallus gallus*	liver	ERR348573	100	110,458,976	20,095,817	1182	X52392
	Upupidae	*Upupa epops*	retina and cochlea	SRR3203224	126	90,142,570	4,448,201	1550	KT356220
	Falconidae	*Falco tinnunculus*	retina and cochlea	SRR3203231	126	100,655,654	6,448,806	1403	NC_011307
	Accipitridae	*Aegypius monachus*	retina and cochlea	SRR3203236	126	95,683,290	5,999,094	1818	KF682364
Mammalia	Hominidae	*Homo sapiens*	brain	SRR611068	101	80,651,654	8,716,424	49,201	NC_012920
	Muridae	*Mus musculus*	heart and kidney	SRR3655009	151	34,241,696	4,141,929	770	NC_005089
	Elephantidae	*Elephas maximus*	blood	SRR2911072	100	70,104,696	2,570,995	1531	DQ316068
	Bovidae	*Bos taurus*	liver	SRR3168807	100*	48,278,779	2,277,754	5086	AF492351
Amphibia	Pipidae	*Xenopus tropicalis*	brain	SRR1189558	101	31,605,646	1,092,532	706	NC_006839

^a^Most are paired-end reads, but a value with an asterisk indicates single-end reads

^b^Number of paired-end (except for *B. taurus*) RNA-Seq reads

^c^Sum of numbers of independent polyA-containing reads for all major polyadenylation sites (forward and reverse paired-end reads derived from a common cDNA fragment were merged for the independent counting)

^d^An accession number for *T. tachydromoides* relates to the mtDNA sequence determined for the RNA-sequenced individual. Accession numbers for the other species correspond to the mtDNA sequences of a conspecific or congeneric individual obtained from the DDBJ/ENA/GenBank database, which was used to infer the mtDNA sequence of the RNA-sequenced individual. An unpublished mtDNA sequence (Yamada, C., unpublished data) was referred to for *T. carolina*

### Mitochondrial DNA sequencing

A nearly complete mtDNA sequence of *T. tachydromoides* was reported by Kumazawa [22] (18,245 bp, DDBJ accession number AB080237). We synthesized 35 primers dispersed in the entire mtDNA based on this information (Additional file 1: Table S1). Total DNA was extracted using a DNeasy Tissue Kit (Qiagen) from the same individual of *T. tachydromoides* as used for the RNA-Seq. Using the total DNA as a template, we amplified ~1.5 kb DNA fragments with the above-mentioned primers. PCR was conducted by 30 cycles of denaturation at 98 °C for 5 s, annealing at 55 °C for 15 s, and extension at 72 °C for 20 s. SpeedStar HS DNA polymerase (Takara) was used for the PCR. Amplified products were subjected to the ExoSAP-IT (Affymetrix) treatment and dye termination reaction with a Big Dye Terminator version 3.1 Cycle Sequencing Ready Reaction Kit (Life Technologies). Sequences obtained with the Applied Biosystems Genetic Analyzer 3500 Sequencer were edited and assembled using Sequencer ver. 4.8 (Gene Codes) to give rise to a nearly complete mtDNA sequence.

With respect to vertebrate species other than *T. tachydromoides*, we only deduced their mtDNA sequences based on the RNA-Seq data under the assumption that no systematic base changes occur in mitochondrial RNAs by the RNA editing [23, 24]. Known RNA editings in vertebrate mitochondria only alter some bases in tRNAs (see, e.g., [25, 26]) although mRNA editings have been reported from some invertebrates ([27] and refs. Therein). We first downloaded a complete mtDNA sequence of the same or closely related species from the DDBJ/EMBL/GenBank databases (see Table 1 for accession numbers). The RNA-Seq reads were then mapped to the mtDNA sequence using Bowtie 1.1.2 [28]. The mapping information was output to a sam file, from which a consensus sequence at all mapped sites was obtained by SAMtools [29]. Some unmapped gaps were usually found in low-read-coveraged and/or repetitive regions of the MNCR and, very rarely, rRNA genes. They were replaced by the corresponding sequences of the reference mtDNA of the same or closely related species.

### Identification of polyadenylation sites

We wrote a script named polyA_seq.pl (Additional file 2) in order to search for reads containing a part of polyA sequences at the 3′ end of RNAs. The polyA_seq.pl searches for oligoA-containing reads that have a more-than-7-base stretch of adenosine at the 3′ end or a more-than-7-base stretch of thymidine at the 5′ end. In light of the Illumina next-generation sequencing error after homopolymer sequences [30], we modified the script to allow occurrence of an extra terminal base, after the stretch of A (i.e., G, C, or T) or before the stretch of T (i.e., G, C, or A). When the number of consecutive A (or T) bases was set to be more than 7, the number of detected polyA-containing reads necessary for identifying major polyadenylation sites was significantly reduced (Additional file 3: Figure S1). When it was set to be lower than 7, falsely positive reads (i.e., those containing the A or T stretches as a part of mtDNA sequences) increased considerably. We thus fixed the number of consecutive As/Ts to be 7 in the script.

The blastn search (E-value cutoff: 1e-5) using BlastStation-Local 64 (TM Software) was conducted with the oligoA-containing reads as a database and the determined/deduced mtDNA sequence of the individual used for the RNA-Seq as a query. Reads that showed the matching in the E-value criterion were assembled with Sequencher 4.8 (Gene Codes) into contigs. After the oligoA parts were removed from the 3′ end of individual contigs, they were assembled to the mtDNA sequence. When a stretch of A follows the assembled sites in the mtDNA sequence, this contig represents the false positive of the polyadenylation signal (i.e., the stretch of A derived from a part of the mtDNA sequence). These falsely positive contigs were removed and the polyadenylation sites were identified based on the assembled sites of the remaining contigs and singletons. OligoA-containing reads constituting these remaining contigs and singletons are called polyA-containing reads hereafter. Polyadenylation sites were identified as the last base encoded in the mtDNA just before the stretch of A starts. However, when the range of a neighboring tRNA can clearly specify a processing site that leaves one or two adenosine residues at the 3′ end of the processed RNA, this information was used to specify the polyadenylation sites. In this study positions at which the polyA attachment was confirmed with 10 or more independent polyA-containing reads were considered as major polyadenylation sites. The proportions of mtDNA-derived reads and polyA-containing reads in total RNA-Seq reads were rather heterogeneous across the species (Table 1), and we did not use criteria normalized by the total read numbers for assigning the major polyadenylation sites.

Confirmation of the polyadenylation sites by 3′ RACE experiments was conducted using a SMARTer™ RACE cDNA Amplification Kit (Clontech). After amplifying 3′ end portions of targeted genes using a specific forward primer for the gene and a common reverse primer provided in the kit, amplified products were recovered from an agarose gel using a MinElute Gel Extraction Kit (Qiagen) and ligated to a plasmid vector by a Mighty TA-cloning Kit (Takara). After cloning with NEB 10-beta competent cells (New England Biolabs), several colonies were subjected to the colony PCR and subsequent sequencing.

Secondary structures of mRNA sequences around the polyadenylation sites (a range covering 100 bp before and after the polyadenylation site) were sought by the RNA Secondary Structure option of DNASIS-MAC version 3.5 (Hitachi) and RNAstructure (http://rna.urmc.rochester.edu/RNAstructureWeb/) in the default conditions.

### Inference of mRNA units

The major polyadenylation sites described above specify 3′ end positions of individual mt-mRNAs preceding the polyA tail. We attempted to gain information on ranges of these mt-mRNAs by approximating their 5′ ends using the RNA-Seq data.

The RNA-Seq reads were subjected to the trimming of low-quality (less than 10 in the phred quality score; [31]) and short (less than 20 bp) reads with SolexaQA [32]. The in-house blastn search (E-value cutoff: 1e-5) was then conducted with the mtDNA sequence of an RNA-sequenced individual as a query and the RNA-Seq reads after trimming as a database. Based on sequence IDs of the reads that matched the blastn conditions, we obtained nucleotide sequences of the mtDNA-derived reads in the fasta format using the blastdbcmd program of the blast+ package.

These mtDNA-derived reads were mapped to the mtDNA sequence of the RNA-sequenced individual with Bowtie 1.1.2. The mapped information was output to a sam file, which was then converted to a bam file sorted by position using SAMtools. The bam file was analyzed using the genomeCoverageBed software of the bedtools v2.26.0 utilities [33] to calculate the frequency of mapped reads for each mtDNA site. The ratio of the read frequency at a mtDNA site relative to that at a − 10 nucleotide site (i.e., a slope of the read frequency by a 10-nucleotide window) was plotted against the position number of a − 5 nucleotide site. The −5 nucleotide site at which the ratio increases to 1.5 or more was considered as approximation of the 5′ ends of mRNAs. In a region with relatively low read frequencies, stochastic fluctuations of read frequencies may lead to false signals in the increase of the slope. Thus, positions with less than 1500 read frequencies were excluded from this procedure.

A rationale behind this approximation is as follows. The reads mapped to coding regions of protein genes greatly outnumber those mapped to other regions including the MNCR and tRNA genes. This is because short RNAs of less than 100 nucleotides (e.g., mature tRNA molecules) and any RNAs without the polyA tail do not contribute to the RNA-Seq reads in the standard RNA-Seq condition. Thus, an mRNA should have contiguously high frequencies of mapped reads throughout its range and the 5′ end position of mRNAs may be approximated around a site where the slope of mapped read frequencies considerably (1.5 times or more in our analytical setting) increases.

Using these procedures, we approximated ranges of H-strand-transcribed mRNAs, which usually count to be ten because ATP8/ATP6 and ND4L/ND4 are two pairs of genes that make a dicistronic mRNA [1, 34]. The ND6 mRNAs lacking the polyadenylation at their 3′ termini [11, 12] may have been excluded in the polyA-RNA enrichment stage using the oligo-dT beads. We thus could not infer ranges of the L-strand-transcribed ND6 mRNAs, except for birds (see below), in this study.

## Results

### RNA and mtDNA sequencing for *T. tachydromoides*

**Fig. 2:**
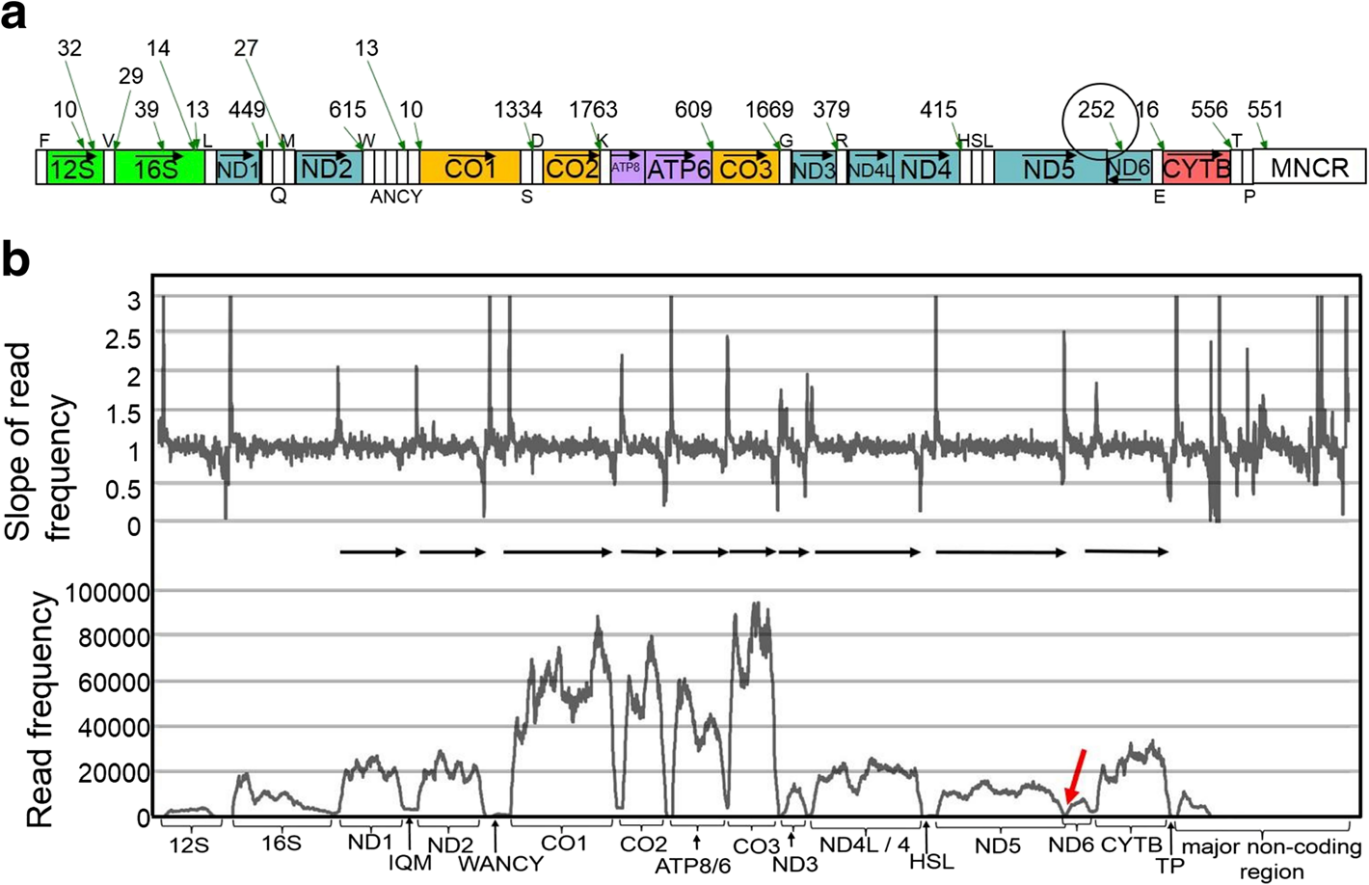
Characterization of *Takydromus tachydromoides* mt-mRNAs using RNA-Seq reads. **a** Gene organization of *T. tachydromoides* mtDNA with major polyadenylation sites. Major polyadenylation sites for H-strand-transcribed and L-strand-transcribed RNAs supported by 10 or more independent (forward and reverse paired-end reads derived from a common cDNA fragment were merged for the counting) polyA-containing reads are shown over and under the column, respectively, with the number of the supporting polyA-containing reads. Note that no major polyadenylation sites were found for L-strand transcripts of *T. tachydromoides*. Changes in mRNA polyadenylation sites as compared to the human [7] are highlighted by a circle. Refer to the legend of Fig. 1 for gene abbreviations and other details. **b** Mapping of RNA-Seq reads to *T. tachydromoides* mtDNA. At the bottom, frequency of the RNA-Seq reads covering each site of the 17,923-bp mtDNA sequence is plotted in the vertical direction. In the horizontal direction, ranges for protein (from a start codon to a stop codon) and rRNA genes are shown along with tRNA gene cluster regions (IQM, WANCY and HSL). A sharp decline of read frequencies, which corresponds to the new polyadenylation site for ND5 mRNA (see text), is indicated by a red arrow. At the top, the slope of the mapped read frequencies as measured by sliding a 10-nucleotide window is plotted in the vertical direction. Ranges for H-strand-transcribed mRNAs estimated are illustrated by horizontal arrows

We obtained 101-bp paired-end reads of cDNA fragments with the Illumina HiSeq™ 2000 Sequencer, starting from *T. tachydromoides* liver mRNAs (53,655,734 reads in total as counted for both directions; DDBJ Sequence Read Archive accession number DRR072216; Table 1). We then determined a nearly complete (17,923 bp) mtDNA sequence of a *T. tachydromoides* individual used for the RNA-Seq (DDBJ accession number LC101816). Because a 65-bp sequence repeated at least 10 times in the 5′ half of the MNCR, it was difficult to obtain the complete mtDNA sequence. We did not find any site that showed heteroplasmy in the determined 17,923-bp mtDNA sequence. The gene arrangement of the mtDNA (Fig. 2a) was identical to that of a different *T. tachydromoides* individual [22], as well as those of the human [1] and many other vertebrates [2, 3].

The blastn search was conducted with the mtDNA sequence of the RNA-sequenced individual as a query and the RNA-Seq reads as a database. As a result, 3,627,027 reads (6.8%) (Table 1) were identified as mtDNA-derived reads (DDBJ Sequence Read Archive accession number DRZ007676). Namely, nearly 7% of the total RNA-Seq reads corresponded to transcripts from the mtDNA. These reads were then mapped to the 17,923-bp mtDNA sequence with Bowtie 1.1.2 (Fig. 2b; Additional file 1: Table S2). When nucleotide sequences were compared between the mtDNA sequence and a consensus of the mapped read sequences, there was no distinct base between them in coding regions of twelve H-strand-encoded protein genes (data not shown).

### Polyadenylation sites in *T. tachydromoides*

Using the polyA_seq.pl program, 181,063 oligoA-containing reads were found among 53,655,734 RNA-Seq reads for *T. tachydromoides*. The blastn search subsequently found 18,554 mtDNA-derived oligoA-containing reads (Additional file 3: Figure S1) and the inspection of assembling sites to the mtDNA identified 9753 mtDNA-derived polyA-containing reads having the A or T stretch that was not present in the mtDNA sequence (data not shown). Figure 2a shows major polyadenylation sites for *T. tachydromoides* mitochondrial RNAs at which ten or more independent polyA-containing reads supported occurrence of the polyadenylation. When the A/T stretch was removed, 8795 independent polyA-containing reads were assembled next to these major polyadenylation sites (Table 1). The remaining 958 polyA-containing reads were assembled to other positions where less than 10 reads (only one in many cases; see Additional file 3: Figure S2) were assembled. At all of the major polyadenylation sites, the polyA sequence was attached to the 3′ ends of L-strand sequences (i.e., H-strand transcripts).

Figure S2 in Additional file 3 shows minor polyadenylation sites for *T. tachydromoides* mitochondrial RNAs, next to which 2–9 polyA-containing reads were assembled when the stretch of A was removed. Except for numerous minor polyadenylation sites within 12S and 16S rRNA genes, there were only 9 minor polyadenylation sites in the other regions: 6 sites on H-strand transcripts and 3 closely neighboring sites on L-strand ones. Because all the 6 sites on H-strand transcripts were located in the middle of protein coding regions, they did not appear to correspond to polyadenylation sites for any H-strand-transcribed mRNAs. In addition, these minor polyadenylation sites were based on only a few polyA-containing reads and their biological significance was less certain than that of the major polyadenylation sites described above.

Six out of the twelve H-strand-encoded protein genes do not have a complete stop codon in the mtDNA sequence, and addition of the polyA tail to the 3′ end of processed mRNAs generates a UAA stop codon (Additional file 1: Table S3). For example, the ND2 gene terminates with T just before the tRNA^Trp^ gene (Additional file 1: Table S2). The precursor ND2 mRNA should terminate with U immediately after being cut at the 5′ end of the tRNA^Trp^. Then, the polyadenylation generates a UAA stop codon for the ND2 mRNA. There were 615 polyA-containing reads that supported the polyadenylation at this position (Fig. 2a). Similarly, 1334 polyA-containing reads supported the polyadenylation at the 3′ end of CO1 mRNA after being cut at the 5′ end of the tRNA^Asp^. For mRNAs of the ND1, CO2, ATP8/ATP6, CO3, ND3, ND4L/ND4 and CYTB genes, polyA-containing reads with the numbers shown in Fig. 2a supported the polyadenylation right after the 3′ end of their reading frames. These polyadenylation sites for *T. tachydromoides* (Fig. 2) were common to those for the human (Fig. 1).

Two possible polyadenylation sites were identified for *T. tachydromoides* ND5 mRNAs. One was at the 5′ end of the CYTB gene supported by 16 polyA-containing reads (Fig. 2a). This is the same polyadenylation site as for mammalian ND5 mRNAs [7]. The second site was identified 113 bp downstream of the ND5 stop codon within the antisense sequence of the ND6 gene, which was supported by 252 polyA-containing reads (Fig. 2a). We conducted 3′ RACE experiments to confirm the occurrence of the polyadenylation at the second site (Additional file 3: Figure S3). Taken together, major polyadenylation sites were identified for all ten mature mRNAs corresponding to twelve H-strand-encoded protein genes.

There were several major polyadenylation sites that were not located at the 3′ end vicinity of protein genes (Fig. 2a). For example, six major polyadenylation sites were found in the 12S and 16S rRNA gene regions although they were supported by relatively few numbers of reads (10–39 reads). Moreover, 551 polyA-containing reads were assembled to the repetitive region within the MNCR. Because this repetitive region consisted of at least 10 times repeated arrays of a 65-bp sequence, we did not exactly know which of these repeat units actually allowed the polyadenylation in transcribed mitochondrial RNAs. In this regard, it is noticeable that a polyadenylated long (375 nt) noncoding RNA that terminated in the MNCR was reported for the Atlantic cod with a potential regulatory role in coordinating transcriptions from both strands [35].

The major polyadenylation sites were also present in the tRNA gene cluster regions of IQM and WANCY (Fig. 2a). In the IQM cluster, 27 reads included polyA sequences that started just before the tRNA^Met^ gene. Among the 27 polyA-containing reads, 8 paired reads extended their 5′ ends to the ND1 coding region (Additional file 3: Figure S4). This suggests that RNAs that gave rise to these 8 reads, at least, extended from the ND1 coding region. A dominant polyadenylation site for the ND1 mRNA exists at the 3′ end of ND1 coding region (Fig. 2a). Thus, the polyadenylation site inside the IQM region may represent a secondary polyadenylation site for the ND1 mRNA. Alternatively, polyadenylation may have occurred to semi-stable processing intermediates of immature transcripts.

The WANCY cluster included two major polyadenylation sites. One is within an antisense sequence of the tRNA^Cys^ gene (23 bp from the 3′ end of the antisense sequence) supported by 13 polyA-containing reads and another is within an antisense sequence of the tRNA^Tyr^ gene (4 bp before the CO1 gene) supported by 10 polyA-containing reads. When 5′ ends of paired reads for these polyA-containing reads were examined, none of them extended their 5′ ends to the ND2 coding region (Additional file 3: Figure S4). Thus, what structural and functional features were held by RNAs having the polyA tail inside the WANCY region remains elusive.

### Inference of mRNA units in *T. tachydromoides*

Based on the rationale described in Methods, we tried to approximate ranges of H-strand-transcribed mt-mRNAs for *T. tachydromoides*. The frequency of mapped reads at individual mtDNA sites and the slope of the frequencies measured by sliding a 10-nucleotide window (Fig. 2b) clearly suggested units of ten H-strand-transcribed mt-mRNAs.

For example, the mapped read frequency considerably (1.5 or more in the slope) increased around base position 5290 (as conventionally numbered from the 5′ end of the tRNA^Phe^ gene) near a start codon for the CO1 gene (position 5285), stayed at high levels throughout its coding region, and rapidly decreased near the polyadenylation site (position 6898) (Additional file 1: Table S2). The CO1 mRNA unit was thus inferred to range from the vicinity of its start codon to the polyadenylation site (Fig. 2b). This range was similar to those found for mammalian CO1 mRNAs [7, 11, 17, 34]. In the same way the mRNAs for ND1, ND2, CO2, ATP8/ATP6, CO3, ND3, ND4L/ND4 and CYTB genes were inferred to range from the vicinity of their start codons to the polyadenylation sites near their stop codons (Fig. 2b; Additional file 1: Table S2).

It should be noted that this procedure did not precisely identify the 5′ end of mt-mRNAs. The approximated position for the 5′ end was 5–24 nt (16 nt in average) larger than the starting position of the mt-mRNA coding regions (Additional file 1: Table S2). This is because RNA-Seq reads mapped to 5′ and 3′ ends of mRNAs are relatively scarce when they are obtained with the random-priming reverse transcription [36, 37]. In addition, RNA-Seq reads derived from immature polycistronic transcripts, though they are present in much smaller amounts than mature mRNAs (see Discussion), obscure the 5′ end location of mature mRNAs in the mapped read profiles.

The 3′ end of ND5 mRNA is located far downstream of its stop codon in mammals (i.e., at the 5′ end of the CYTB gene) [7]. In *T. tachydromoides*, however, there was a sharp decline of read frequencies in the middle of the ND6 coding region (see a red arrow in Fig. 2b), where there was a *de novo* polyadenylation site (Fig. 2a). Thus, a primary ND5 mRNA for *T. tachydromoides* has a distinct range from the mammalian counterparts. It starts from the vicinity of the ND5 start codon and ends 113 bp downstream of the ND5 stop codon (Fig. 2b). We were unable to infer a range for ND6 mRNA because of the presumable lack of its polyadenylation (Fig. 2a), as has been found for mammalian ND6 mRNAs [9].

### Polyadenylation sites in other species

**Fig. 3:**
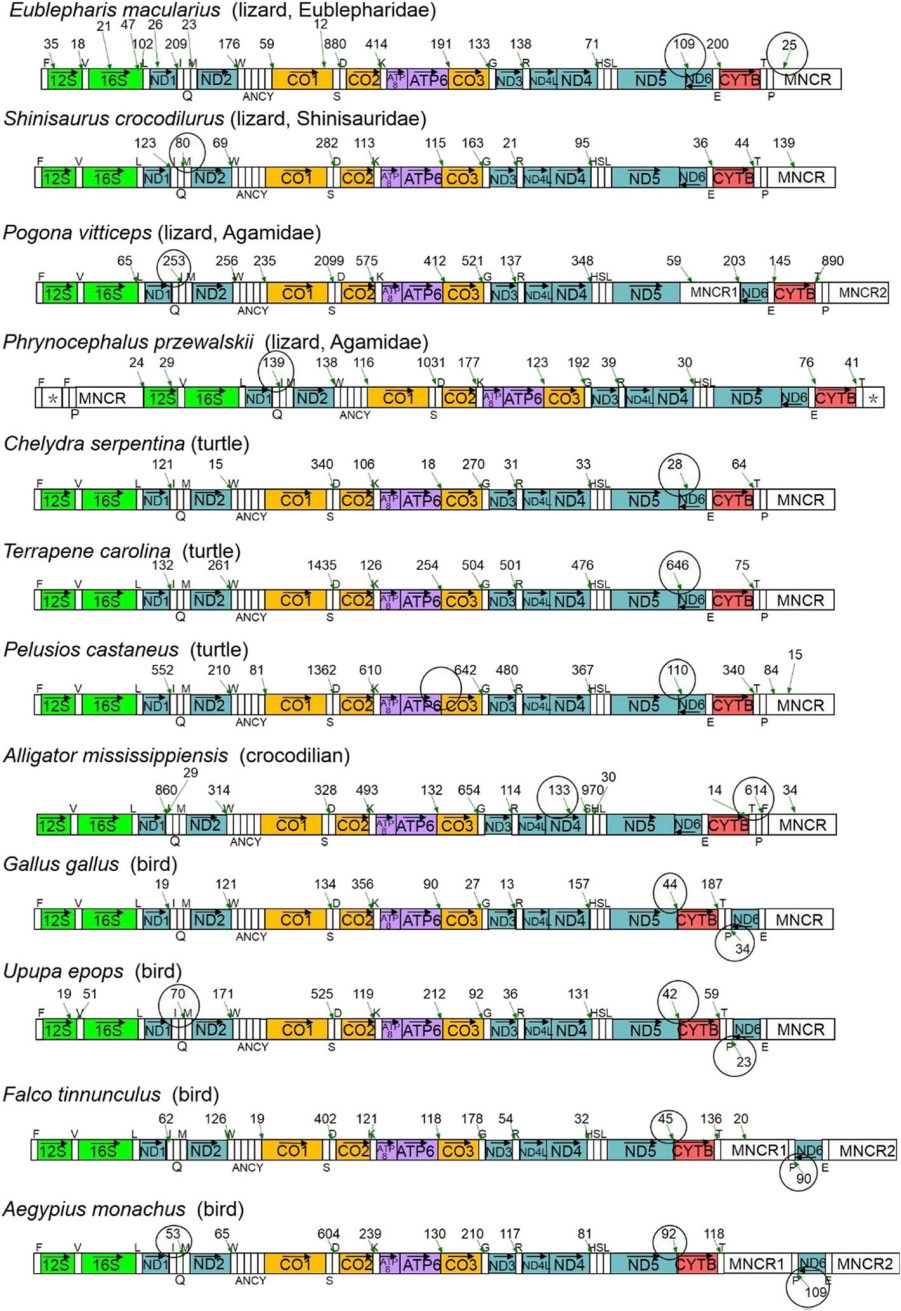
Major polyadenylation sites for twelve tetrapod species in which the polyadenylation profile differed from that of the human. Refer to the legend of Fig. 1 for gene abbreviations and the use of colors and that of Fig. 2 for ways to show major polyadenylation sites with numbers of supportive polyA-containing reads. Asterisks in *P. przewalskii* show truncated major noncoding regions. Changes in mRNA polyadenylation sites as compared to the human noted in text are highlighted by a circle. Note that polyadenylation sites for *P. vitticeps* ND5 mRNA could not be surely identified due to the insertion of an extra copy of MNCR. Also note that there was a minor polyadenylation site 242 bp downstream of the ND5 stop codon as supported by 6 polyA-containing reads in *A. mississippiensis* (data not shown) and this site may function as a polyadenylation site for the ND5 mRNA

We downloaded RNA-Seq data for other 23 species (Table 1) and used them to identify polyadenylation sites and infer units of ten H-strand-transcribed mRNAs by the same method as used for *T. tachydromoides*, except that mtDNA sequence of the RNA-sequenced individual was not experimentally determined but deduced from the RNA-Seq data. In 11 species among them (including *H. sapiens*) polyadenylation sites for the ten mt-mRNAs were located in similar positions as found for the human (Additional file 3: Figure S5). The remaining 12 species had some changes in the polyadenylations sites from those in the human, although five of them retained the typical mtDNA gene arrangements (Fig. 3).

### Lizards

A gecko lizard (*E. macularius*) had the typical mtDNA gene arrangements but its polyadenylation sites for ND5 and CYTB mRNAs were found to be different from those in the human (Fig. 3). Two possible polyadenylation sites were identified for the ND5 mRNAs just like in *T. tachydromoides*. One was at the 5′ end of the CYTB gene (the same position as in the human) supported by 200 polyA-containing reads. The second polyadenylation site was identified 78 bp downstream of the ND5 stop codon, which was supported by 109 polyA-containing reads (Fig. 3). A polyadenylation site for the human CYTB mRNA occurs at the 5′ end of the tRNA^Thr^ gene (Additional file 3: Figure S5; [7]). However, this site disappeared and an alternative polyadenylation site supported by 25 polyA-containing reads existed inside the MNCR in *E. macularius* (Fig. 3). The extension of the CYTB mRNA into the MNCR was supported by the mapped read frequency profile in which there was no recognizable decline of the mapped read frequency at the conventional polyadenylation site (Additional file 3: Figure S6). The new polyadenylation site in MNCR corresponded to a base within the 62-bp repetitive unit but we were unable to specify which repetitive unit did allow the polyadenylation.

Two lizard species from the family Agamidae (*P. vitticeps* and *P. przewalskii*) commonly have a rearranged QIM (instead of IQM in the typical gene organization) tRNA gene order (Fig. 3). Polyadenylation sites for the ND1 mRNAs of these agamids were shifted approximately 70 bp downstream, i.e., from the immediate 3′ end of the ND1 gene to between tRNA^Gln^ and tRNA^Ile^ genes. This change appears to have responded to the IQM to QIM rearrangement and the polyA addition to the 3′ end of RNAs processed at the 5′ end of tRNA^Ile^ can account for the polyadenylation profile.

### Turtles

In three turtle species (*C. serpentina, P. castaneus* and *T. carolina*) having the typical mtDNA gene arrangements, the far polyadenylation site for the ND5 mRNA located next to the CYTB gene was entirely lost and new polyadenylation sites were created at closer positions to the ND5 stop codons (Fig. 3). The polyadenylation site for *C. serpentina* ND5 mRNA was identified 287 bp downstream of the ND5 stop codon within the antisense sequence of the ND6 gene with 28 polyA-containing reads. The polyadenylation site for *T. carolina* ND5 mRNA was identified 70 bp downstream of the ND5 stop codon within an intergenic spacer between ND5 and ND6 genes, which was supported by 646 polyA-containing reads. Finally, *P. castaneus* had a polyadenylation site for the ND5 mRNA 11 bp downstream of the ND5 stop codon also within the intergenic spacer between ND5 and ND6 genes, and 110 polyA-containing reads supported this result.

One of the turtle species (*P. castaneus*) had a striking change in the range of ATP8/ATP6 and CO3 mRNAs. A polyadenylation site for the ATP8/ATP6 mRNA usually occurs immediately 3′ to the ATP6 gene for other species (Figs. 1–3; Additional file 3: Figure S5). However, there is no polyadenylation site at this location in *P. castaneus* (Fig. 3). A mapped read frequency profile for this species (Additional file 3: Figure S7) showed continuously high levels of the frequency from the ATP8 gene to CO3 gene, suggesting that ATP8/ATP6 and CO3 mRNAs are now combined to share a tricistronic mRNA. This is a unique characteristics in *P. castaneus* shared by none of the other vertebrate species examined in this study.

### Alligator

In the crocodilian *A. mississippiensis*, there was a novel polyadenylation site 647 bp downstream of the ND4L stop codon with 133 supportive polyA-containing reads. This is unusual because ND4L and ND4 share a polyadenylation site for a dicistronic mRNA for other species (Figs. 1–3; Additional file 3: Figure S5). In *A. mississippiensis*, this dicistronic mRNA likely occurs as supported by 970 polyA-containing reads (Fig. 3). Because there is a partial decline of mapped read frequency at the new polyadenylation site 647 bp downstream of the ND4L stop codon (Additional file 3: Figure S8), the monocistronic ND4L mRNA may co-exist partly.

In addition, there was a novel polyadenylation site supported by 614 polyA-containing reads for the *A. mississippiensis* CYTB mRNA just before the tRNA^Phe^ gene (Fig. 3), which had been moved to the 5′ end of the MNCR by a gene rearrangement [38, 39]. The canonical polyadenylation site at the 5′ end of the tRNA^Thr^ gene partially remained with only 14 supportive polyA-containing reads. We confirmed that a large fraction (39%) of paired reads of the 614 polyA-containing reads had their 5′ ends in the CYTB gene (data not shown), supporting that *A. mississippiensis* CYTB mRNA has its primary polyadenylation site just before the tRNA^Phe^ gene.

### Birds

Bird mtDNAs share a rearranged gene organization in which genes for ND6 and tRNA^Glu^ are translocated to the proximity of the MNCR [40]. As a result, two H-strand-encoded genes, ND5 and CYTB, are juxtaposed without intervening tRNA genes. Polyadenylation sites for the ND5 mRNAs of four bird species (*G. gallus, U. epops, F. tinnunculus* and *A. monachus*) occurred immediately after the ND5 stop codon in response to this gene rearrangement (Fig. 3). A notable finding on the polyadenylation sites for these birds was that they clearly included polyadenylation sites for ND6 mRNAs (Fig. 3). The ND6 is the only protein gene encoded by the L-strand and the polyadenylation to the ND6 mRNAs was not detected from any nonavian taxa we examined (Figs. 2 and 3; Additional file 3: Figure S5).

It is also noteworthy that polyadenylation sites for ND1 mRNAs varied among the four birds. The ND1 polyadenylation sites usually occur immediately before the tRNA^Ile^ gene in most taxa examined in this study including two birds (*G. gallus* and *F. tinnunculus*). However, the ND1 polyadenylation site occurred immediately before the tRNA^Met^ gene in two other birds (*U. epops* and *A. monachus*) although gene arrangements around the IQM tRNA gene cluster were common among the four birds (Fig. 3). Also, in a lizard *S. crocodilurus*, two possible polyadenylation sites were identified for the ND1 mRNAs (Fig. 3). One was immediately before the tRNA^Ile^ gene (the same position as in the human) with 123 supportive polyA-containing reads. The second polyadenylation site was identified immediately before the tRNA^Met^ gene with 80 supportive polyA-containing reads.

## Discussion

### Analysis of mitochondrial mRNAs with RNA-Seq data

Studies on gene expression from vertebrate mtDNAs have been carried out using model organisms (e.g., human and mouse) by molecular biological characterization of transcripts of some target genes, as well as proteins interacting with the mtDNAs and transcripts ([6, 7, 9, 34, 41] and refs. Therein). This approach provided numerous progresses but had a shortcoming in the difficulty of simultaneously monitoring the state of individual transcripts. The high-throughput RNA-Seq has opened a way to overcome this problem and the characterization of organellar transcripts using this technology is considered to be promising [42]. To our knowledge, the high-throughput approach has been utilized for characterizing mt-mRNAs in particular metazoan taxa (see, e.g., [11, 17, 18, 43–45]) but it has rarely been applied to their comparisons among diverse taxonomic groups. In this study, we attempted to elucidate structural diversity of mt-mRNAs among sauropsids efficiently and comprehensively by the high-throughput method.

Because populations of multiple RNA molecules are simultaneously analyzed in the high-throughput approach, some precautions may be necessary to interpret the results. First, it is difficult to judge whether an RNA-Seq read was derived from a mature mRNA or an immature transcript. In this regard, we estimated the relative abundance of the immature transcripts using the Reads Per Kilobase per Million mapped reads (RPKM) [46] for the tRNA gene cluster regions (IQM, WANCY and HSL) vs. coding regions of ten H-strand-transcribed mRNAs (Additional file 1: Tables S3 and S4). Because mature tRNAs having much less than 100 nucleotides and no polyA sequences at their ends do not basically contribute to the RNA-Seq, RNA-Seq reads including the tRNA gene sequences likely derived from the immature transcripts before being processed at the 5′ and 3′ ends of tRNAs. Based on this assumption, we roughly estimated that 1.6–9.0% of the reads assigned to H-strand-transcribed mRNAs were derived from the immature transcripts (Additional file 1: Table S4). We thus consider that the majority of RNA-Seq reads reflects the status in mature mt-mRNAs.

Second, standard RNA-Seq experiments do not retain information on the strand of RNAs from which the RNA-Seq library was constructed. However, directional RNA-Seq is not necessary for the purpose of identifying polyadenylation sites at the 3′ end of mt-mRNAs because coding strands for the mt-mRNAs are obvious based on the mtDNA sequences. When we analyzed directional RNA-Seq data for the human (SRR3151753), we could identify similar major polyadenylation sites (Additional file 3: Figure S9) as found, using the non-directional RNA-Seq, for another human individual (SRR611068; Additional file 3: Figure S5). In the directional RNA-Seq data, 703,613 mtDNA-derived reads were identified from 29,236,394 sense-strand reads. Among them, 690,892 reads (98.2%) were L-strand sequences and only 12,271 reads (1.8%) were H-strand ones (Additional file 3: Figure S9). When ranges for ten mt-mRNAs were considered individually, the contribution from the L-strand transcripts stayed at the low level (0.9–4.1%; Additional file 3: Figure S9). We also observed similar patterns of very low contributions from the antisense RNA sequences in directional RNA-Seq data for the mouse (SRR1976596) and the American bison (SRR1659067)(data not shown).

Third, in order to map RNA-Seq reads to individual genes or mRNAs, a reference mtDNA sequence is needed. However, RNA-Seq data in public databases are not usually accompanied by mtDNA sequences of the RNA-sequenced individual. The mtDNA sequence of another individual of the same species or of the same genus is often available in the databases but mismatches at polymorphic sites could lower the mapping efficiency and bias the mapping profile. To address this problem, we deduced the mtDNA sequence of the RNA-sequenced individual based on a consensus sequence of the RNA-Seq reads mapped to the conspecific (or congeneric) mtDNA sequence. Similar methods were reported by Nabholz et al. [23] and Tian and Smith [24] using avian and algal mitochondrial genomes, respectively. This procedure also worked well in the present study although gaps were usually present in the MNCR. As demonstrated for *T. tachydromoides* in this study (see the first section of Results), no major RNA editing sites have been found in protein-coding genes of vertebrate mtDNAs, justifying the use of the deduced mtDNA sequence for subsequent analyses.

In our study, RNAs used for the RNA-Seq mostly came from liver for reptilian taxa but those for non-reptilian taxa were derived from different organs including liver, brain, blood, retina and cochlea (Table 1). Although the variation of polyA lengths among different cell lines could occur [10], we did not know any report that clearly showed changes in major polyadenylation sites for mt-mRNAs between organs. We therefore had an assumption that the major polyadenylation sites do not change by organ, sex, age and environment (such as nutritional status), which should be examined in details by identifying major polyadenylation sites with diverse samples of a species in future.

### Timing and mechanisms of the polyadenylation site changes for ND5 mRNAs

**Fig. 4:**
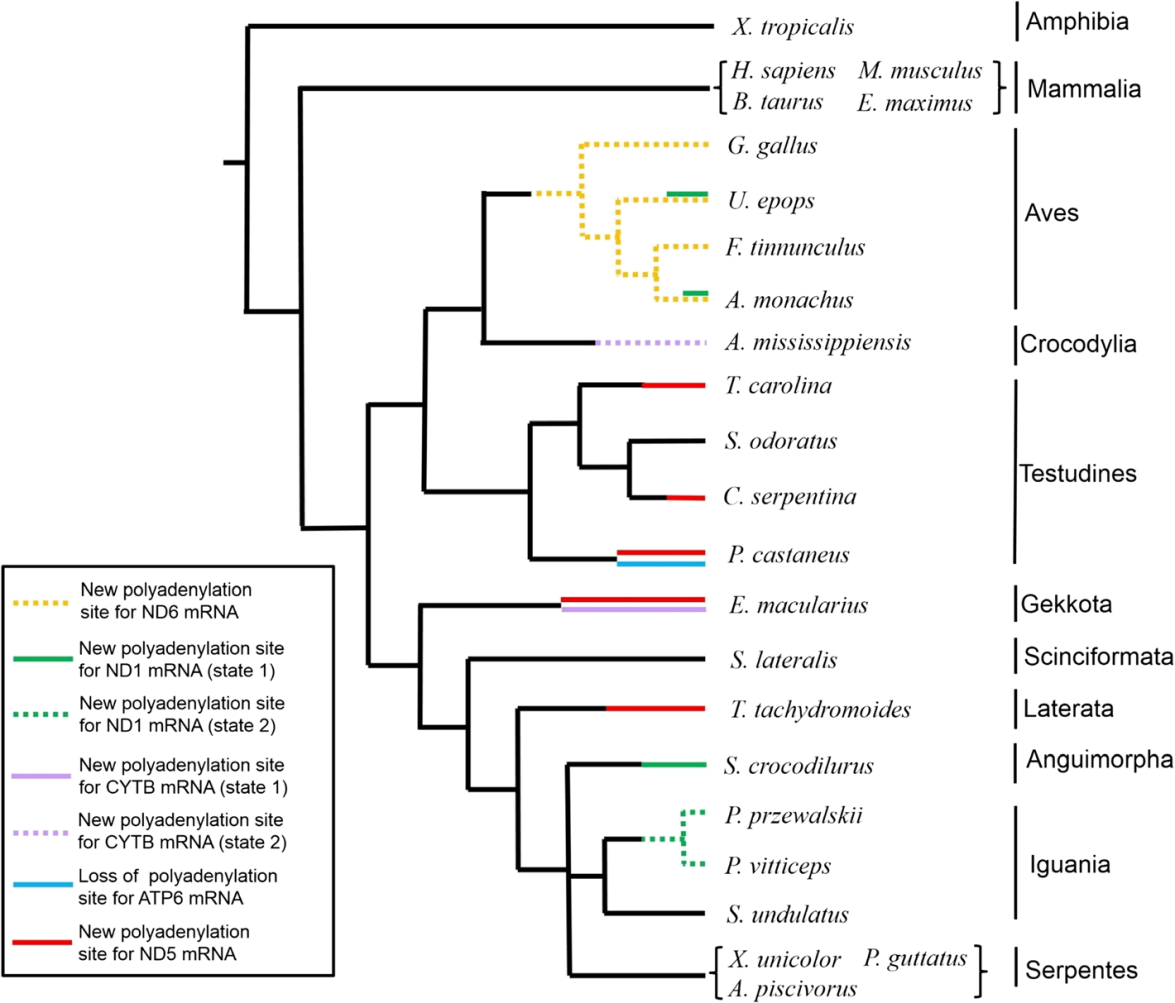
Evolution of polyadenylation profiles in sauropsid mitochondrial mRNAs. Colored lineages show the presence of new polyadenylation sites as estimated by the parsimony criterion based on well-established phylogenetic relationships [56]. Dotted lineages show the occurrence of new polyadenylation sites possibly linked with mtDNA gene rearrangements. New polyadenylation sites are considered in this figure when the number of polyA-containing reads that support them is more than a half of that supporting the polyadenylation sites found for the human. New polyadenylation sites for the ND1 mRNA have two states, which existed without gene rearrangement in the IQM tRNA gene cluster (state 1) and with the IQM-to-QIM gene rearrangement (state 2). New polyadenylation sites for the CYTB mRNA also have two states, which existed without gene rearrangement around the CYTB gene (state 1) and with a rearrangement of the tRNA^Phe^ gene near the CYTB gene (state 2)

In this study, we examined the variability of sauropsid mt-mRNAs with respect to their ranges and polyadenylation sites in comparison with other tetrapods (mammals and amphibians). As a result, different polyadenylation profiles from that for the human were found in 12 of 23 tetrapods. We estimated the timing for changes in the polyadenylation sites by the parsimony criterion based on well-established phylogenetic relationships (Fig. 4).

The polyadenylation site for the human ND5 mRNA is located far away from the ND5 stop codon just before the CYTB gene, creating a long 3′ UTR for the ND5 mRNA (Fig. 1). In this study, we found new polyadenylation sites for the ND5 mRNA in 5 species (*T. tachydromoides, E. macularius, P. castaneus, C. serpentina,* and *T. carolina*) (Figs. 2 and 3). In three turtles among them, the polyadenylation site at the 5′ end of the CYTB gene completely disappeared and new polyadenylation sites were created 11 bp, 70 bp, and 287 bp after the ND5 stop codon in *P. castaneus*, *T. carolina* and *C. serpentina*, respectively (Fig. 3). In two lizards, the mammalian polyadenylation site at the 5′ end of the CYTB gene remained partially and new polyadenylation sites were created 113 bp and 78 bp after the ND5 stop codon in *T. tachydromoides* (Fig. 2) and *E. macularius* (Fig. 3), respectively. The new polyadenylation sites in *C. serpentina* and the two lizards are located in different positions of the ND6 antisense coding sequence (data not shown). Based on the phylogenetic relationships of Fig. 4, we consider that these new ND5 polyadenylation sites arose independently on individual lineages. In this regard, it should be noted that the 3′ UTR of the ND5 mRNA is also severely truncated in codfishes [18].

With respect to the functional consequences of shortening the 3′ UTR of ND5 mRNAs, the long 3′ UTR for ND5 mRNAs was suggested to act like a long noncoding RNA that interacts with the L-strand-transcribed ND6 mRNA for an unknown mechanism of gene expression regulation [47, 48]. Mammalian mitochondria tightly control the respiratory activity through the regulation of ND5 polypeptide synthesis and its integration into the respiratory complex I [49, 50]. In species that truncated the 3′ UTR of ND5 mRNA, the interaction between this 3′ UTR and the ND6 mRNA may be lost or weakened. This may potentially affect the binding of trans-acting factors, if any, to the ND6 mRNA, leading to a somewhat different way of the post-transcriptional control of gene expression. However, the human ND6 mRNA was suggested to have long (500–600 nt) 3′ UTR sequences [12] and this may serve for the interaction between ND5 and ND6 mRNAs even though the 3′ UTR of ND5 mRNA is severely truncated. Another interpretation of the shortened 3′ UTR of ND5 mRNAs in some species is that the 3′ UTR part may have become an independent long noncoding RNA molecule found by Rackham et al. [48].

In order to consider evolutionary processes for the changes of the ND5 polyadenylation sites, the RNA secondary structure was inferred near the new polyadenylation sites. We found a tRNA-like secondary structure for *T. tachydromoides* (Additional file 3: Figure S10) but not for the other species (*E. macularius, P. castaneus, C. serpentina,* and *T. carolina*). In this secondary structure, base pairings resembling the acceptor stem and the T stem of vertebrate mitochondrial tRNAs [51] were conserved while structures equivalent to the anticodon and D arms were not. Crystal structures of bacterial RNase Z (an endonuclease whose mitochondrial counterpart is responsible for cutting the 3′ end of tRNAs; [52, 53]) complexed with tRNA substrates indicated that this RNase binds a tRNA-precursor substrate from the acceptor-stem terminal to the T arms but not the anticodon arm [54]. Thus, the secondary structure shown in (Additional file 3: Figure S10) may be recognized by mitochondrial RNase Z to cleave a precursor transcript at the *de novo* polyadenylation site, followed by the polyadenylation to the 3′ end by the mitochondrial polyA polymerase [14].

### Timing and mechanisms of the polyadenylation site changes for other mRNAs

A polyadenylation site was found at the 3′ end of the ND6 mRNAs of four birds while it was not detected for the ND6 mRNAs of nonavian taxa (Figs. 2 and 3; Additional file 3: Figure S5). It was therefore suggested that the polyadenylation site for the L-strand-transcribed ND6 mRNA was acquired in a common ancestor of birds (Fig. 4). Birds commonly share a mtDNA gene rearrangement that transferred genes for the ND6 and tRNA^Glu^ to the vicinity of the MNCR, resulting in the juxtaposition of genes for the ND5 and CYTB, as well as for the tRNA^Pro^ and ND6 [40]. It seems likely that the gene rearrangement caused the change of the polyadenylation profiles. In birds, the tRNA^Pro^ probably serves as a punctuation signal on the precursor L-strand transcript for the subsequent polyadenylation at this site. Polyadenylation for the ND5 mRNA occurs immediately after the ND5 gene (i.e., immediately before the CYTB gene) in contrast with mammals that have a very long 3′ UTR for the ND5 mRNAs (Fig. 1; Additional file 3: Figure S5). This is also likely to be due to the gene rearrangement.

In two lizard species from Agamidae, a polyadenylation site for the ND1 mRNA shifted approximately 70 bp downstream from the immediate 3′ end of the ND1 gene to the junction between tRNA^Gln^ and tRNA^Ile^ genes (Fig. 3). This change likely occurred in response to the IQM-to-QIM tRNA gene rearrangement that occurred in the common ancestor of Agamidae and Chamaeleonidae [55](Fig. 4). Because the tRNA^Gln^ gene is encoded by the L-strand, an antisense tRNA^Gln^ sequence may not function as a punctuation signal on the precursor H-strand transcript, enabling the polyadenylation for the ND1 mRNA to occur next to the 5′ end of the H-strand-encoded tRNA^Ile^ gene.

**Fig. 5:**
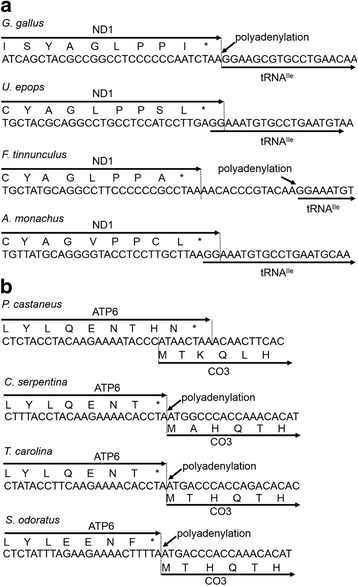
Nucleotide and amino acid sequences at the junction between ND1 and tRNA^Ile^ genes in four birds **a** and between ATP6 and CO3 genes in four turtles **b**. The relevant mtDNA sequences deduced for the RNA-sequenced individual are shown together with translated amino acid sequences. An asterisk shows the position of a stop codon. In **a**, the ND1 and tRNA^Ile^ genes of *U. epops* and *A. monachus* are overlapped without a polyadenylation site at the 5′ end of the tRNA^Ile^ gene. In the other two birds, there is no gene overlap and the polyadenylation occurs at the 5′ end of the tRNA^Ile^ gene. In **b**, ATP6 and CO3 genes of *P. castaneus* are overlapped without a polyadenylation site at this junction. In the other turtles, there is no gene overlap and the processing and subsequent polyadenylation at the junction can create stop codons for the ATP8/ATP6 mRNAs

In the above-mentioned cases of the polyadenylation site changes, as well as the new polyadenylation site for *A. mississippiensis* CYTB mRNA outlined earlier, their relationship to mtDNA gene rearrangements seems reasonable. However, the present study also found polyadenylation profile changes that do not appear to be linked to gene rearrangements. In two birds (*U. epops* and *A. monachus*), the ND1 polyadenylation site shifted from the 5′ end of the tRNA^Ile^ gene to the 5′ end of the tRNA^Met^ gene without involving a gene rearrangement in the IQM tRNA gene cluster (Fig. 3). We examined the boundary sequence of the ND1 and tRNA^Ile^ genes and found a 2-bp overlap of these genes for *U. epops* and *A. monachus* but not for *G. gallus* and *F. tinnunculus* (Fig. 5a). Thus, the shift of the polyadenylation site for the ND1 mRNA seems to be associated with the ND1-tRNA^Ile^ gene overlap. Based on phylogenetic relationships of birds [56], *U. epops* and *A. monachus* do not have a sister-group relationship. We therefore consider that the change in the ND1 mRNA polyadenylation site occurred independently on lineages leading to *U. epops* and *A. monachus* (Fig. 4). Alternatively, the polyadenylation site change may have occurred in the common ancestor of *U. epops*, *F. tinnunculus* and *A. monachus* with subsequent reversal to the original state on a lineage leading to *F. tinnunculus*. Interestingly, a lizard (*S. crocodilurus*) had two major polyadenylation sites at the 5′ ends of the tRNA^Ile^ gene (159 reads) and the tRNA^Met^ gene (105 reads) (Fig. 3). This may represent a transient state of the polyadenylation site change.

In a pleurodiran turtle *P. castaneus*, a polyadenylation site for the ATP8/ATP6 mRNA appears to have been lost (Fig. 3; Additional file 3: Figure S7). Because there is no other taxa that showed the signature of the polyadenylation site disappearance at this position, this change likely occurred on a pleurodiran lineage leading to *P. castaneus* (Fig. 4). The nucleotide sequence at the boundary of ATP6 and CO3 genes of *P. castaneus* indicated that cutting the mRNA in the vicinity of the 5′ end of CO3 gene and subsequent addition of a polyA tail could not create an in-frame stop codon for ATP6 translation (Fig. 5b). In addition, an overlap of ATP6 and CO3 genes was found for *P. castaneus* but not for the other taxa including three turtles (Fig. 5b and data not shown). Thus, the disappearance of the polyadenylation site for the ATP8/ATP6 mRNA is linked with the ATP6-CO3 gene overlap. In human, two gene overlaps are known: one between ATP8 and ATP6 genes and the other between ND4L and ND4 genes [1]. Both of the gene overlaps are associated with the sharing of an mRNA between overlapping genes [34]. Efficient translation of partly overlapping genes with a dicistronic mRNA was demonstrated using the in vitro translation system of tobacco chloroplasts [57]. These evidence corroborates that the ATP8, ATP6, and CO3 genes are translated via a tricistronic mRNA in *P. castaneus*.

## Conclusions

The present study used the high-throughput RNA sequencing approach to show that major polyadenylation sites in sauropsid mt-mRNAs have evolved either with or without the association of mtDNA gene rearrangements. Some changes in the polyadenylation sites were linked with gene overlaps in the mtDNA. Considerable truncation of the 3′ UTR sequences of the ND5 mRNAs occurred independently in multiple lineages and this truncation may have modified the function of the 3′ UTR as a long noncoding RNA. The efficient and comprehensive characterization of mt-mRNAs will contribute to broaden our understanding of their structural and functional evolution.

## Additional files

Additional file 1: Table S1. Primers to determine the mtDNA sequence of *T. tachydromoides*. **Table S2.** Gene organization of *T. tachydromoides* mtDNA and positions of the 5′ end approximation and major polyadenylation sites assigned for H-strand-transcribed mRNAs. **Table S3.** Numbers of coding region lengths, assigned reads, and RPKMs of *T. tachydromoides* mt-mRNAs and tRNA gene clusters. **Table S4.** RPKM values for H-strand-transcribed mt-mRNAs and tRNA gene cluster regions in different taxa. (XLSX 947 kb)

Additional file 2: A Perl script named polyA_seq.pl used to find oligoA-containing reads. (TXT 1 kb)

Additional file 3: Figure S1. Read numbers obtained using the polyA_seq.pl program in different conditions. **Figure S2.** Minor polyadenylation sites for *T. tachydromoides* mitochondrial RNAs. **Figure S3.** Confirmation of the new polyadenylation site for *T. tachydromoides* ND5 mRNA by 3′ RACE. **Figure S4.** 5′ end mapping of *T. tachydromoides* cDNA fragments with polyadenylation at their 3′ end inside mitochondrial tRNA genes. **Figure S5.** Major polyadenylation sites for 11 species in which the polyadenylation profile was similar to that of the human. **Figure S6.** Characterization of *E. macularius* mt-mRNAs using RNA-Seq reads. **Figure S7.** Characterization of *P. castaneus* mt-mRNAs using RNA-Seq reads. **Figure S8.** Characterization of *A. mississippiensis* mt-mRNAs using RNA-Seq reads. **Figure S9.** Characterization of *Homo sapiens* mt-mRNAs using directional RNA-Seq reads (SRR3151753). **Figure S10.** The secondary structure of the L-strand sequence around the *de novo * polyadenylation site for the *T. tachydromoides* ND5 mRNA. (PDF 1437 kb)

## Abbreviations

ATP6 and ATP8: ATP synthase subunits 6 and 8
CO1–3: Cytochrome oxidase subunits I-III
CYTB: Cytochrome *b*
H-strand: Heavy strand
L-strand: Light strand
MNCR: Major noncoding region
mtDNA: Mitochondrial DNA
mt-mRNA: Mitochondrial mRNA
ND1–6 and 4 L: NADH dehydrogenase subunits 1–6 and 4 L
RNA-Seq: RNA sequencing
RPKM: Reads Per Kilobase per Million mapped reads
UTR: Untranslated region

## References

[1] Anderson S, Bankier AT, Barrell BG, de Bruijn MHL, Coulson AR, Drouin J, Eperon IC, Nierlich DP, Roe BA, Sanger F, Schreier PH, Smith AJH, Staden R, Young IG (1981). Sequence and organization of the human mitochondrial genome. Nature.

[2] Bernt M, Braband A, Schierwater B, Stadler PF (2013). Genetic aspects of mitochondrial genome evolution. Mol Phylogenet Evol.

[3] Boore JL (1999). Animal mitochondrial genomes. Nucleic Acids Res.

[4] Kumazawa Y, Miura S, Yamada C, Hashiguchi Y (2014). Gene rearrangements in gekkonid mitochondrial genomes with shuffling, loss, and reassignment of tRNA genes. BMC Genomics.

[5] Montoya J, Christianson T, Levens D, Rabinowitz M, Attardi G (1982). Identification of initiation sites for heavy-strand and light-strand transcription in human mitochondrial DNA. Proc Natl Acad Sci U S A.

[6] Clayton DA (1992). Transcription and replication of animal mitochondrial DNAs. Int Rev Cytol.

[7] Ojala D, Montoya J, Attardi G (1981). tRNA punctuation model of RNA processing in human mitochondria. Nature.

[8] Zhao J, Hyman L, Moore C (1999). Formation of mRNA 3′ ends in eukaryotes: mechanism, regulation, and interrelationships with other steps in mRNA synthesis. Microbiol Mol Biol Rev.

[9] Rorbach J, Minczuk M (2012). The post-transcriptional life of mammalian mitochondrial RNA. Biochem J.

[10] Temperley RJ, Wydro M, Lightowlers RN, Chrzanowska-Lightowlers ZM (2010). Human mitochondrial mRNAs–like members of all families, similar but different. Biochim Biophys Acta.

[11] Mercer TR, Neph S, Dinger ME, Crawford J, Smith MA, Shearwood A-MJ, Haugen E, Bracken CP, Rackham O, Stamatoyannopoulos JA, Filipovska A, Mattick JS (2011). The human mitochondrial transcriptome. Cell.

[12] Slomovic S, Laufer D, Geiger D, Schuster G (2005). Polyadenylation and degradation of human mitochondrial RNA: the prokaryotic past leaves its mark. Mol Cell Biol.

[13] Nagaike T, Suzuki T, Katoh T, Ueda T (2005). Human mitochondrial mRNAs are stabilized with polyadenylation regulated by mitochondria-specific poly(A) polymerase and polynucleotide phosphorylase. J Biol Chem.

[14] Chang JH, Tong L (2012). Mitochondrial poly(A) polymerase and polyadenylation. Biochim Biophys Acta.

[15] Levy S, Schuster G (2016). Polyadenylation and degradation of RNA in the mitochondria. Biochem Soc Trans.

[16] Schuster SC (2008). Next-generation sequencing transforms today’s biology. Nat Methods.

[17] Marková S, Filipi K, Searle JB, Kotlík P (2015). Mapping 3′ transcript ends in the bank vole (*Clethrionomys glareolus*) mitochondrial genome with RNA-Seq. BMC Genomics.

[18] Coucheron DH, Nymark M, Breines R, Karlsen BO, Andreassen M, Jørgensen TE, Moum T, Johansen SD (2011). Characterization of mitochondrial mRNAs in codfish reveals unique features compared to mammals. Curr Genet.

[19] McGaugh SE, Bronikowski AM, Kuo CH, Reding DM, Addis EA, Flagel LE, Janzen FJ, Schwartz TS (2015). Rapid molecular evolution across amniotes of the IIS/TOR network. Proc Natl Acad Sci U S A.

[20] Wu Y, Hadly EA, Teng W, Hao Y, Liang W, Liu Y, Wang H (2016). Retinal transcriptome sequencing sheds light on the adaptation to nocturnal and diurnal lifestyles in raptors. Sci Rep.

[21] Martin M (2011). Cutadapt removes adaptor sequences from high-throughput sequencing reads. EMBnetjournal.

[22] Kumazawa Y (2007). Mitochondrial genomes from major lizard families suggest their phylogenetic relationships and ancient radiations. Gene.

[23] Nabholz B, Jarvis ED, Ellegren H (2010). Obtaining mtDNA genomes from next-generation transcriptome sequencing: a case study on the basal Passerida (Aves: Passeriformes) phylogeny. Mol Phylogenet Evol.

[24] Tian Y, Smith DR (2016). Recovering complete mitochondrial genome sequences from RNA-Seq: a case study of *Polytomella* non-photosynthetic green algae. Mol Phylogenet Evol.

[25] Yokobori S, Pääbo S (1995). tRNA editing in metazoans. Nature.

[26] Börner GV, Mörl M, Janke A, Pääbo S (1996). RNA editing changes the identity of a mitochondrial tRNA in marsupials. EMBO J.

[27] Lavrov DV, Adamski M, Chevaldonne P, Adamska M (2016). Extensive mitochondrial mRNA editing and unusual mitochondrial genome organization in calcaronean sponges. Curr Biol.

[28] Langmead B, Trapnell C, Pop M, Salzberg SL (2009). Ultrafast and memory-efficient alignment of short DNA sequences to the human genome. Genome Biol.

[29] Li H, Handsaker B, Wysoker A, Fennell T, Ruan J, Homer N, Marth G, Abecasis G, Durbin R, 1000 Genome Project Data Processing Subgroup (2009). The sequence alignment/map format and SAMtools. Bioinformatics.

[30] Quail MA, Smith M, Coupland P, Otto TD, Harris SR, Connor TR, Bertoni A, Swerdlow HP, Gu Y (2012). A tale of three next generation sequencing platforms: comparison of Ion Torrent, Pacific Biosciences and Illumina MiSeq sequencers. BMC Genomics.

[31] Ewing B, Hillier L, Wendl MC, Green P (1998). Base-calling of automated sequencer traces using *phred*. I. Accuracy assessment. Genome Res.

[32] Cox MP, Peterson DA, Biggs PJ, Solexa QA (2010). At-a-glance quality assessment of Illumina second-generation sequencing data. BMC Bioinform.

[33] Quinlan AR, Hall IM (2010). BEDtools: a flexible suite of utilities for comparing genomic features. Bioinformatics.

[34] Montoya J, Ojala D, Attardi G (1981). Distinctive features of the 5′-terminal sequences of the human mitochondrial mRNAs. Nature.

[35] Jørgensen TE, Bakke I, Ursvik A, Andreassen M, Moum T, Johansen SD (2014). An evolutionary preserved intergenic spacer in gadiform mitogenomes generates a long noncoding RNA. BMC Evol Biol.

[36] Levin JZ, Yassour M, Adiconis X, Nusbaum C, Thompson DA, Friedman N, Gnirke A, Regev A (2010). Comprehensive comparative analysis of strand-specific RNA sequencing methods. Nat Methods.

[37] Brown SM, Goecks J, Brown SM (2015). RNA sequencing with next-generation sequencing. Next-generation DNA sequencing informatics.

[38] Quinn TW, Mindell DP (1996). Mitochondrial gene order adjacent to the control region in crocodile, turtle, and tuatara. Mol Phylogenet Evol.

[39] Janke A, Arnason U (1997). The complete mitochondrial genome of *Alligator mississippiensis* and the separation between recent Archosauria (birds and crocodiles). Mol Biol Evol.

[40] Desjardins P, Morais R (1990). Sequence and gene organization of the chicken mitochondrial genome. A novel gene order in higher vertebrates. J Mol Biol.

[41] Campbell CT, Kolesar JE, Kaufman BA (2012). Mitochondrial transcription factor A regulates mitochondrial transcription initiation. Biochim Biophys Acta.

[42] Smith DR (2013). RNA-Seq data: a goldmine for organelle research. Brief Funct Genom.

[43] Torres TT, Dolezal M, Schlötterer C, Ottenwälder B (2009). Expression profiling of *Drosophila* mitochondrial genes via deep mRNA sequencing. Nucleic Acids Res.

[44] Neira-Oviedo M, Tsyganov-Bodounov A, Lycett GJ, Kokoza V, Raikhel AS, Krzywinski J (2011). The RNA-Seq approach to studying the expression of mosquito mitochondrial genes. Insect Mol Biol.

[45] Wang H-L, Yang J, Boykin LM, Zhao Q-Y, Li Q, Wang X-W, Liu S-S (2013). The characteristics and expression profiles of the mitochondrial genome for the Mediterranean species of the *Bemisia tabaci* complex. BMC Genomics.

[46] Mortazavi A, Williams BA, McCue K, Schaeffer L, Wold B (2008). Mapping and quantifying mammalian transcriptomes by RNA-Seq. Nat Methods.

[47] Tullo A, Tanzariello F, D’Erchia AM, Nardelli M, Papeo PA, Sbisà E, Saccone C (1994). Transcription of rat mitochondrial NADH-dehydrogenase subunits. Presence of antisense and precursor RNA species. FEBS Lett.

[48] Rackham O, Shearwood AM, Mercer TR, Davies SM, Mattick JS, Filipovska A (2011). Long noncoding RNAs are generated from the mitochondrial genome and regulated by nuclear-encoded proteins. RNA.

[49] Bai Y, Shakeley RM, Attardi G (2000). Tight control of respiration by NADH dehydrogenase ND5 subunit gene expression in mouse mitochondria. Mol Cell Biol.

[50] Chomyn A (2001). Mitochondrial genetic control of assembly and function of complex I in mammalian cells. J Bioenerg Biomembr.

[51] Kumazawa Y, Nishida M (1993). Sequence evolution of mitochondrial tRNA genes and deep-branch animal phylogenetics. J Mol Evol.

[52] Lopez Sanchez MIG, Mercer TR, Davies SMK, Shearwood A-MJ, Nygård KKA, Richman TR, Mattick JS, Rackham O, Filipovska A (2011). RNA processing in human mitochondria. Cell Cycle.

[53] Rossmanith W (2012). Of P and Z: mitochondrial tRNA processing enzymes. Biochim Biophys Acta.

[54] Redko Y, Li de la Sierra-Gallay I, Condon C (2007). When all’s zed and done: the structure and function of RNase Z in prokaryotes. Nat Rev Microbiol.

[55] Macey JR, Larson A, Ananjeva NB, Papenfuss TJ (1997). Evolutionary shifts in three major structural features of the mitochondrial genome among iguanian lizards. J Mol Evol.

[56] Hedges SB, Kumar S (2009). The Timetree of Life.

[57] Yukawa M, Sugiura M (2008). Termination codon-dependent translation of partially overlapping *ndhC-ndhK* transcripts in chloroplasts. Proc Natl Acad Sci U S A.

