# Supplementary material for: Variation and evolution of polyadenylation profiles in sauropsid mitochondrial mRNAs as deduced from the high-throughput RNA sequencing
Source: BMC Genomics. 2017 Aug 29;18:665. doi: 10.1186/s12864-017-4080-0 (PMC5576253; doi:10.1186/s12864-017-4080-0)
Supplement: Supplementary file 3 — Read numbers obtained using the polyA_seq.pl program in different conditions. Figure S2. Minor polyadenylation sites for T. tachydromoides mitochondrial RNAs. Figure S3. Confirmation of the new polyadenylation site for T. tachydromoides ND5 mRNA by 3′ RACE. Figure S4. 5′ end mapping of T. tachydromoides cDNA fragments with polyadenylation at their 3′ end inside mitochondrial tRNA genes. Figure S5. Major polyadenylation sites for 11 species in which the polyadenylation profile was similar to that of the human. Figure S6. Characterization of E. macularius mt-mRNAs using RNA-Seq reads. Figure S7. Characterization of P. castaneus mt-mRNAs using RNA-Seq reads. Figure S8. Characterization of A. mississippiensis mt-mRNAs using RNA-Seq reads. Figure S9. Characterization of Homo sapiens mt-mRNAs using directional RNA-Seq reads (SRR3151753). Figure S10. The secondary structure of the L-strand sequence around the de novo polyadenylation site for the T. tachydromoides ND5 mRNA. (PDF 1437 kb) [file 12864_2017_4080_MOESM3_ESM.pdf]

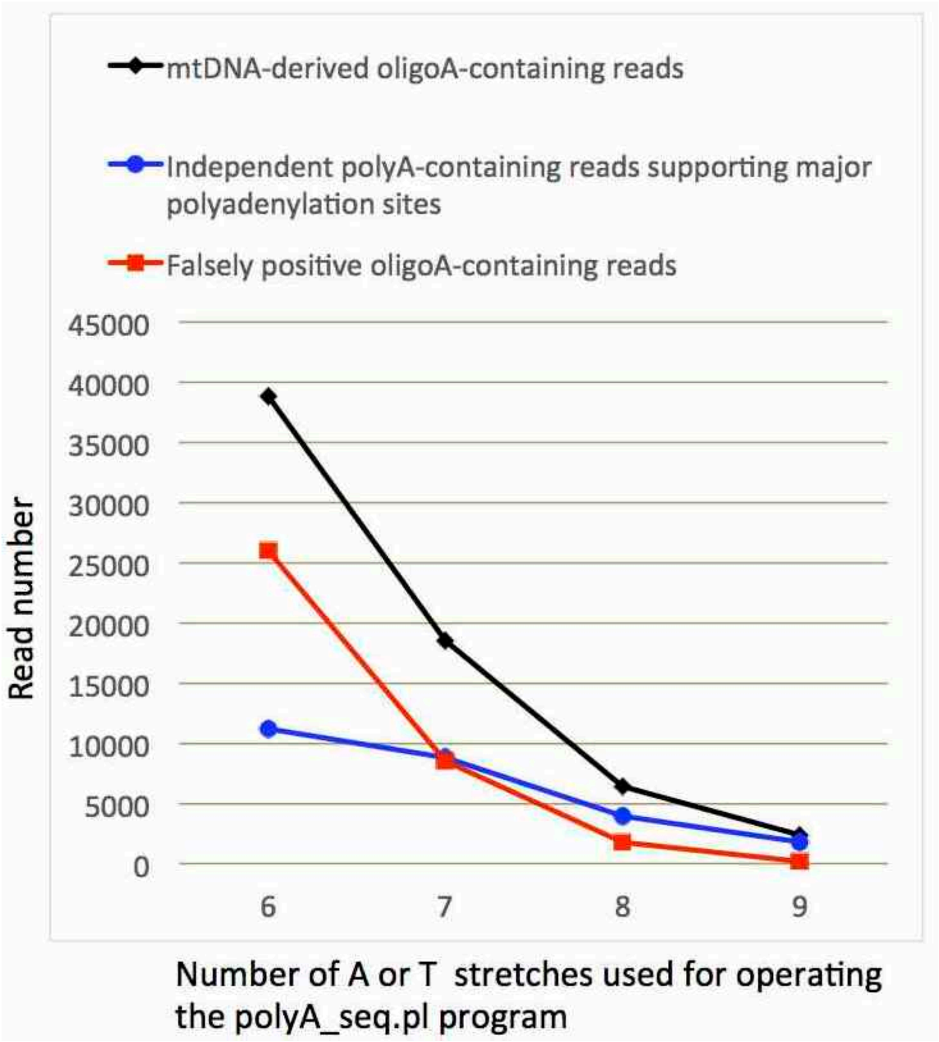


Fig. S1. Read numbers obtained using the polyA_seq.pl program in different conditions. Starting from 53,655,734 RNA-Seq reads of *T. tachydromoides*, the polyA_seq.pl program selected oligoA-containing reads under different settings for the number of A or T stretches at the 3’ or 5’ ends, respectively. The blastn search then identified mtDNA-derived oligoA-containing reads (black diamonds), from which reads supporting major polyadenylation sites were identified (blue circles). The number of falsely positive reads containing the A or T stretches that existed as a part of the mtDNA sequence is shown as red squares. The results indicated steady increase of reads supporting major polyadenylation sites by decreasing the number of A or T stretches. On the other hand, the falsely positive reads rapidly increased by decreasing the number of A or T stretches. Because careful removal of the falsely positive reads was time-consuming and because there was no qualitative difference with respect to the identified major polyadenylation sites between the stretch settings 6 and 7, we fixed the number of A or T stretches as 7 for subsequent analyses.

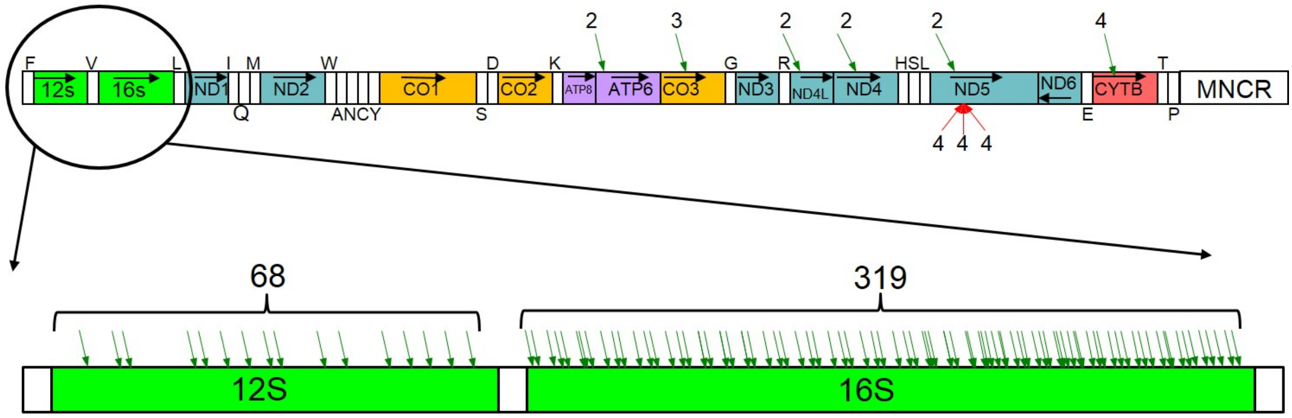


Fig. S2. Minor polyadenylation sites for *T. tachydromoides* mitochondrial RNAs. Polyadenylation sites for H/L-strand transcripts based on 2-9 independent polyA-containing RNA-Seq reads are shown by downward/upward arrows, respectively, with corresponding read numbers. Note that read numbers are not shown at individual sites within 12S and 16S rRNA genes. Refer to the legend of Fig. 1 for gene abbreviations and the use of colors.

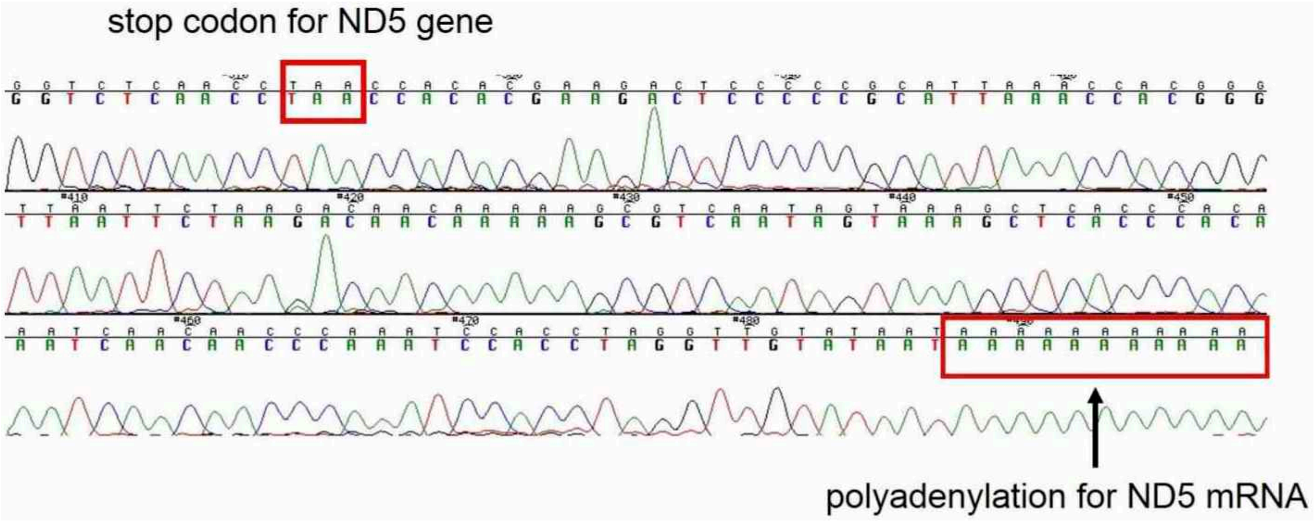


Fig. S3. Confirmation of the new polyadenylation site for *T. tachydromoides* ND5 mRNA by 3' RACE. Amplified products from the 3' RACE were excised from an agarose gel and cloned into an *E. coli* plasmid. Twenty four colonies were subjected to the colony PCR and subsequent sequencing of amplified products to confirm that all the amplified products had the same nucleotide sequence as shown in the figure.

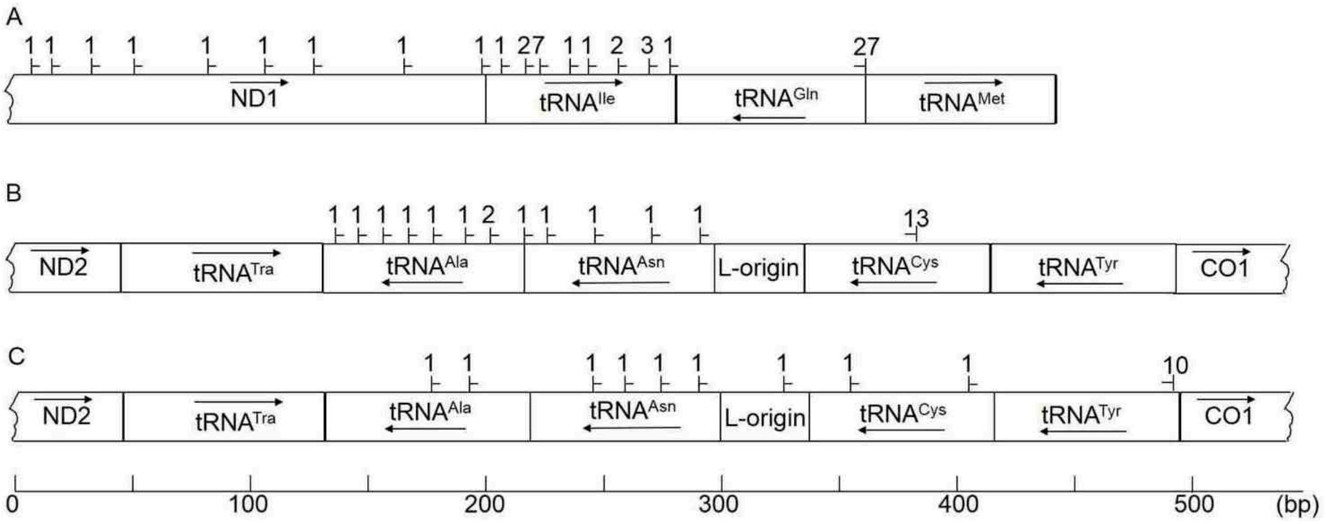


Fig. S4. 5' end mapping of *T. tachydromoides* cDNA fragments with polyadenylation at their 3' end inside mitochondrial tRNA genes. The polyadenylation sites in the $tRNA^{Gln}$ (A), $tRNA^{Cys}$ (B), and $tRNA^{Tyr}$ (C) genes are indicated by "⊣"with numbers of the corresponding polyA-containing reads. The 5' end position of each cDNA fragment is shown by "⊢" with a number of the corresponding reads. Note that, in C, the 5' end of one cDNA fragment was unable to be mapped due to uncertain 5' end sequence. L-origin represents a stem-and-loop structure for the putative light-strand replication origin.

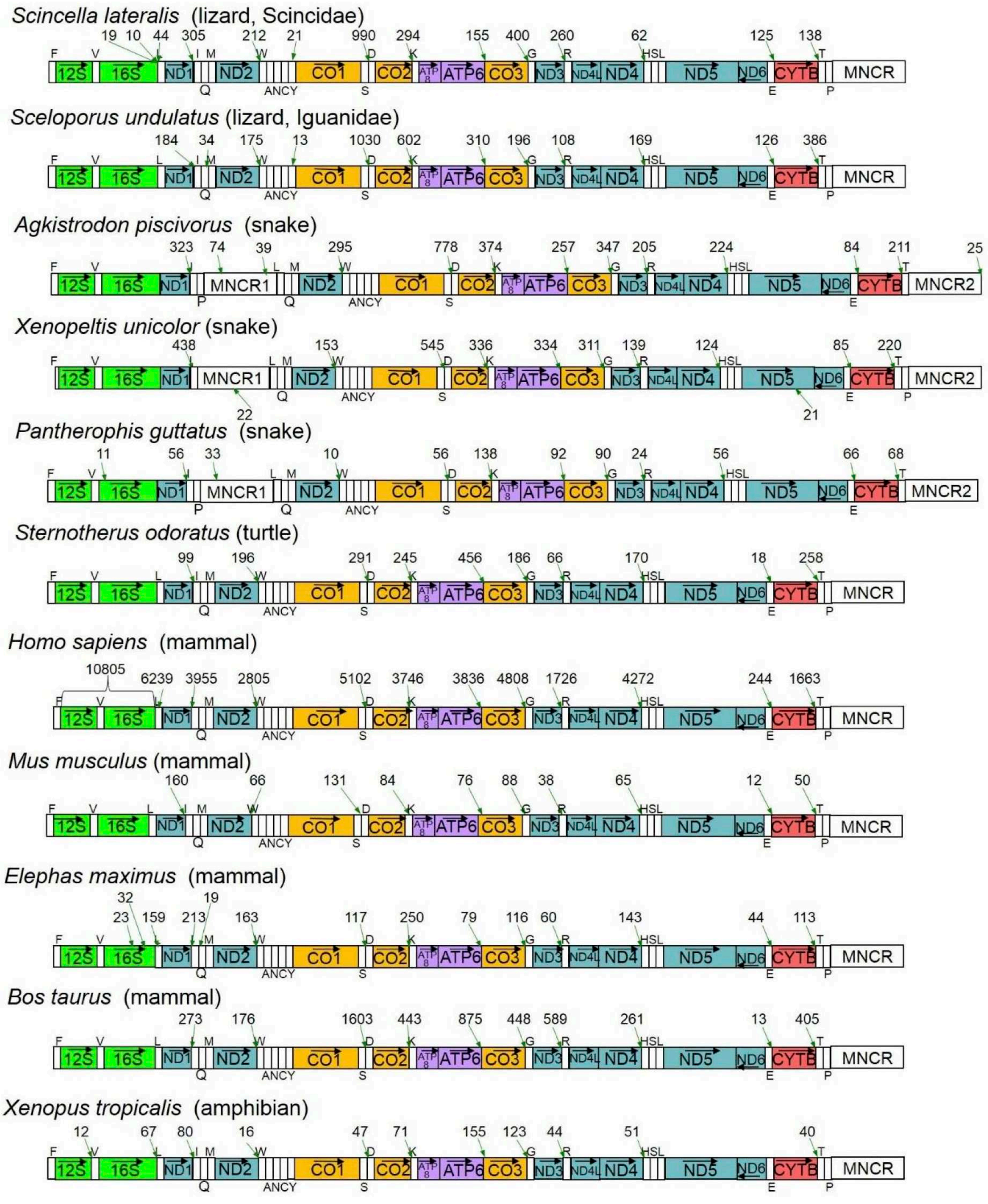


Fig. S5. Major polyadenylation sites for 11 species in which the polyadenylation profile was similar to that of the human. Refer to the legend of Fig. 1 for gene abbreviations and the use of colors and that of Fig. 2 for ways to show major polyadenylation sites with numbers of supportive polyA-containing reads.

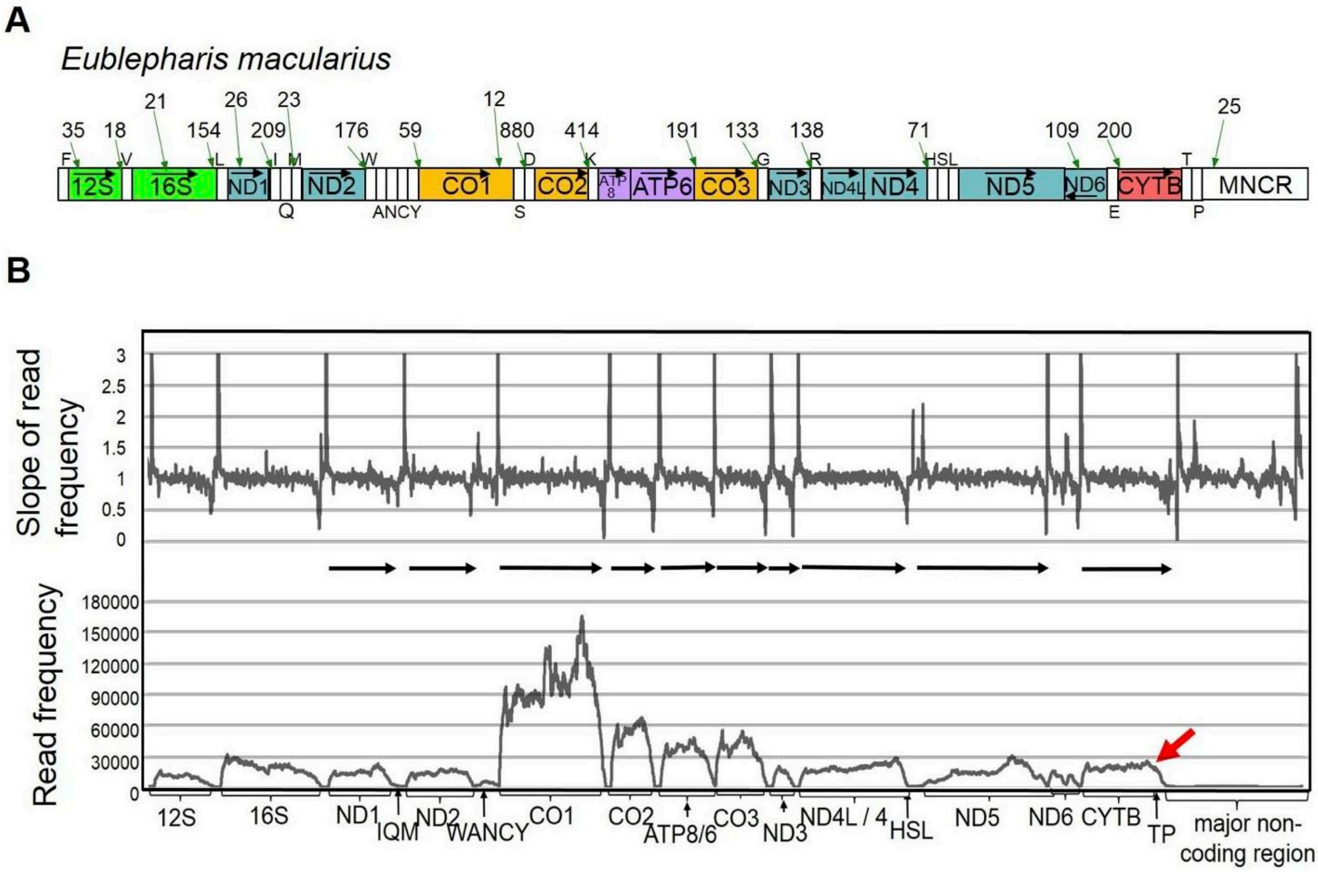


Fig. S6. Characterization of *E. macularius* mt-mRNAs using RNA-Seq reads. (A) Gene organization of *E. macularius* mtDNA with major polyadenylation sites as shown in Fig. 3. (B) Mapping of RNA-Seq reads to *E. macularius* mtDNA. Refer to the legend of Fig. 2 for explanations of the frequency of the mapped RNA-Seq reads (at the bottom) and the slope of the mapped read frequencies (at the top). A red arrow indicates the gene boundary between CYTB and $tRNA^{Thr}$, in which there is no recognizable decline of mapped reads (see text).

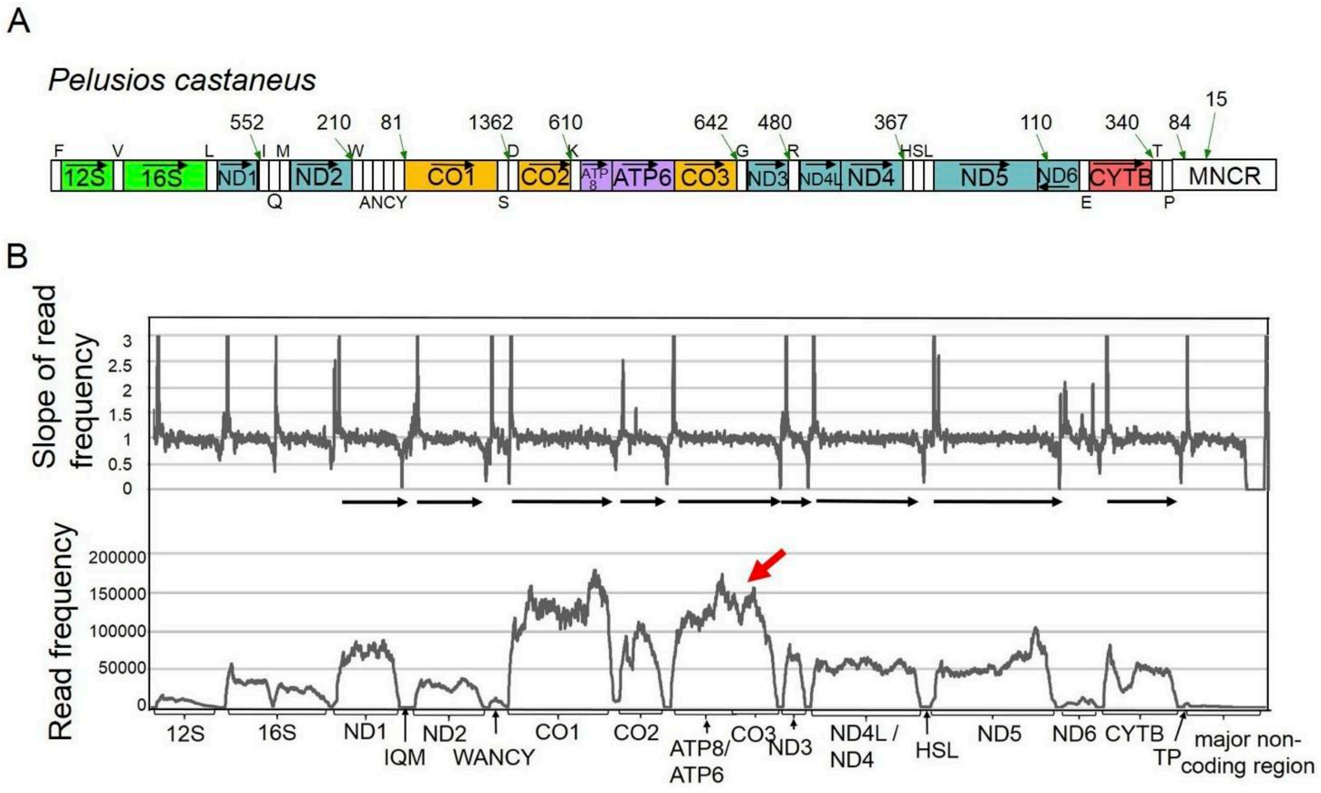


Fig. S7. Characterization of *P. castaneus* mt-mRNAs using RNA-Seq reads. (A) Gene organization of *P. castaneus* mtDNA with major polyadenylation sites as shown in Fig. 3. (B) Mapping of RNA-Seq reads to *P. castaneus* mtDNA. Refer to the legend of Fig. 2 for explanations of the frequency of the mapped RNA-Seq reads (at the bottom) and the slope of the mapped read frequencies (at the top). A red arrow indicates the gene boundary between ATP8/ATP6 and CO3, in which there is no recognizable decline of mapped reads (see text).

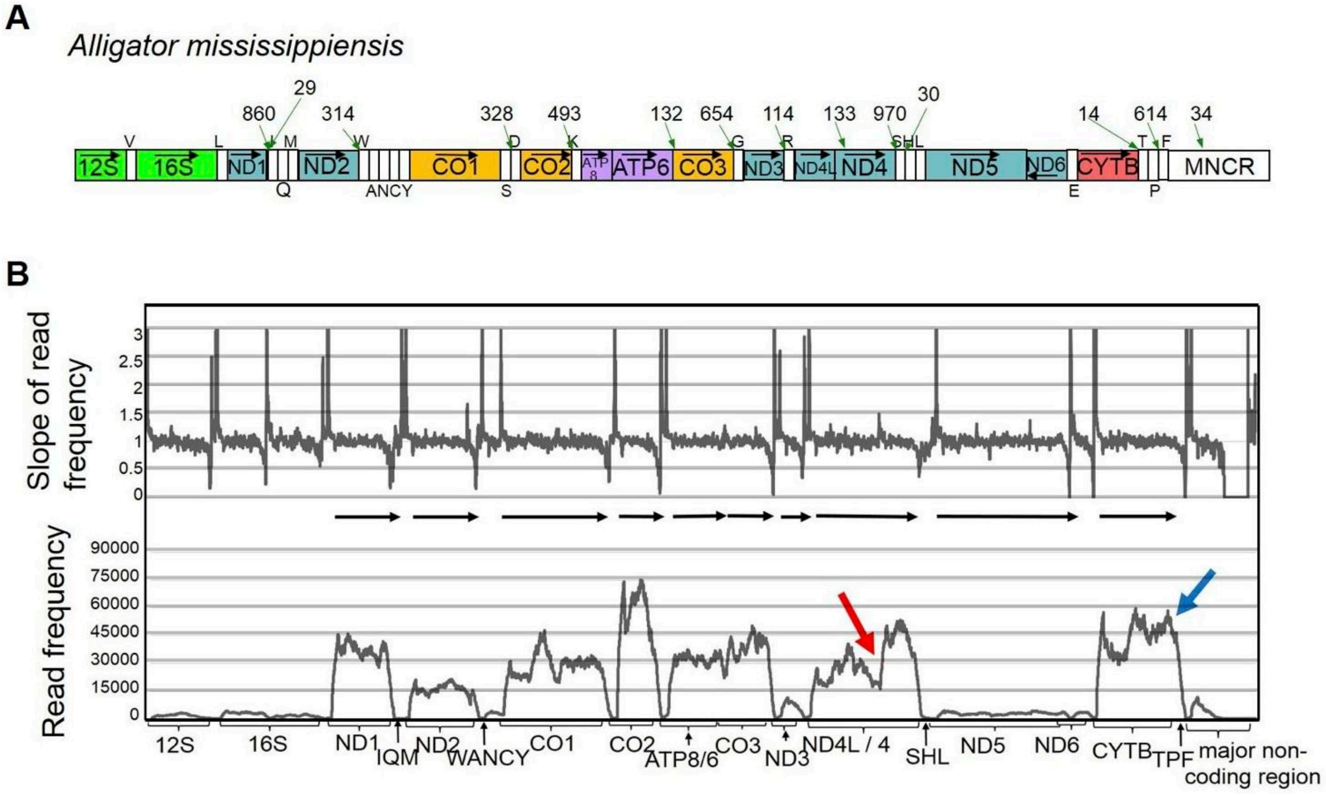


Fig. S8. Characterization of *A. mississippiensis* mt-mRNAs using RNA-Seq reads. (A) Gene organization of *A. mississippiensis* mtDNA with major polyadenylation sites as shown in Fig. 3. (B) Mapping of RNA-Seq reads to *A. mississippiensis* mtDNA. Refer to the legend of Fig. 2 for explanations of the frequency of the mapped RNA-Seq reads (at the bottom) and the slope of the mapped read frequencies (at the top). A red arrow indicates the possible polyadenylation site for the ND4L mRNA, in which there is a partial decline of mapped reads (see text). A blue arrow indicates the gene boundary between CYTB and $tRNA^{Thr}$, in which there is no recognizable decline of mapped reads (see text).

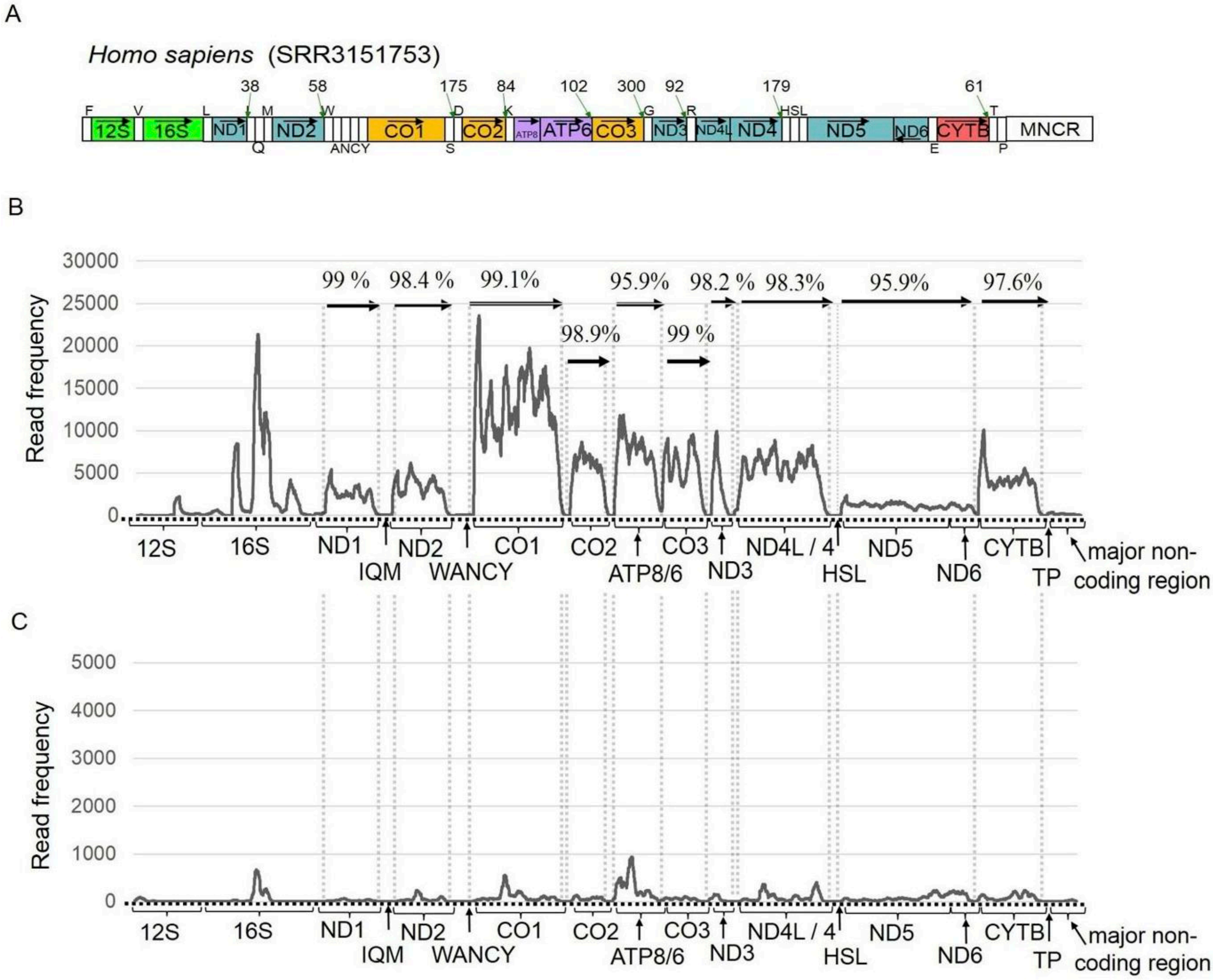


Fig. S9. Characterization of *Homo sapiens* mt-mRNAs using directional RNA-Seq reads (the NCBI SRA database accession No. SRR3151753). (A) Gene organization of *H. sapiens* mtDNA with major polyadenylation sites. (B and C) Frequency of the sense-strand RNA-Seq reads mapped to the human mtDNA sequence for the H-strand transcripts (B) and L-strand transcripts (C). Ranges for H-strand-transcribed mRNAs estimated are illustrated by horizontal arrows and percentages of H-strand transcripts in the corresponding region are shown on them. Refer to the legend of Fig. 2 for other details. The apparent lack of the major polyadenylation site for the ND5 mRNA may be due to smaller polyA-containing reads in SRR3151753 than in SRR611068 (Fig. S5) and shorter polyA sequences attached to the human ND5 mRNA than those attached to other mt-mRNAs (Temperley et al., Biochim. Biophys. Acta 1797: 1081-1085, 2010).

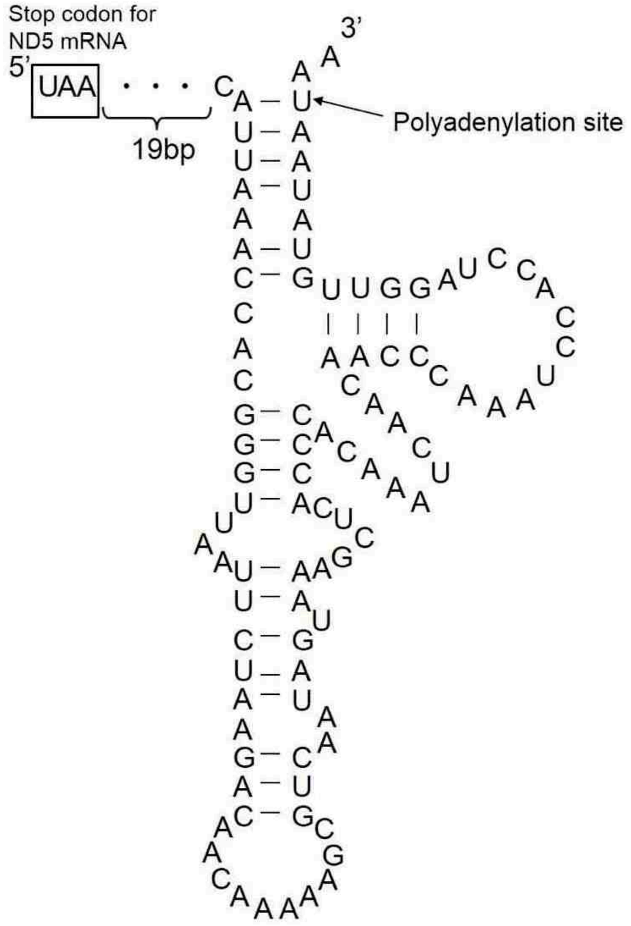


Fig. S10. The secondary structure of the L-strand sequence around the *de novo* polyadenylation site for the *T. tachydromoides* ND5 mRNA. Bars represent possible base pairings.
